# Possibility of Metal Accumulation in Reed Canary Grass (*Phalaris arundinacea* L.) in the Aquatic Environment of South-Western Polish Rivers

**DOI:** 10.3390/ijerph19137779

**Published:** 2022-06-24

**Authors:** Magdalena Senze, Monika Kowalska-Góralska, Katarzyna Czyż, Anna Wondołowska-Grabowska

**Affiliations:** 1 Institute of Animal Breeding, Department of Limnology and Fishery, Wrocław University of Environmental and Life Sciences, ul. Chełmońskiego 38c, 51-630 Wrocław, Poland; monika.kowalska-goralska@upwr.edu.pl; 2 Institute of Animal Breeding, Department of Sheep and Fur Animals Breeding, Wrocław University of Environmental and Life Sciences, ul. Kożuchowska 5A, 51-631 Wrocław, Poland; katarzyna.czyz@upwr.edu.pl; 3 Institute of Agroecology and Plant Production, Wrocław University of Environmental and Life Sciences, Grunwaldzki Sq. 24A, 50-363 Wrocław, Poland; anna.wondolowska-grabowska@upwr.edu.pl

**Keywords:** aquatic plants, copper, cadmium, lead, nickel, zinc, iron, manganese

## Abstract

A four-year research study was conducted on aquatic plants (reed canary grass) growing in the beds of three rivers and their tributaries in Lower Silesia, Poland. Metal contents (Cu, Cd, Ni, Pb, Zn, Fe, Mn) were determined in plant samples, metal accumulation in water (BCF_w_) and sediment (BCF_B_), Metal Pollution Index (MPI) and Enrichment Factor (EF) were calculated. The highest contents of copper, lead, nickel and cadmium were found in reed canary grass sampled from the Nysa Szalona River. The highest values were recorded for zinc in the Bystrzyca River, and for iron and manganese in the Strzegomka River. The series of metals were as follows: Nysa Szalona and Strzegomka: Cd < Ni < Pb < Cu < Zn < Mn < Fe, Bystrzyca: Cd < Ni < Cu < Pb < Zn < Mn < Fe. Throughout the study period, the lowest values of metals in plants were recorded in 2015 and 2018, and the highest in 2017. The general picture of MPI in aquatic plants is arranged in the series Bystrzyca < Strzegomka < Nysa Szalona. These values classify the studied material at a high level of pollution in all rivers. In the comparison of the two extreme sites, i.e., source–mouth, higher values were found at the mouth of the reservoir, which suggests that metals move with the water current and accumulate more with the direction of the river flow, which is most likely a consequence of the influence of the catchment area as the source of metals. The series of EF enrichment factor values were as follows: Bystrzyca—Ni < Cd < Fe < Cu < Zn < Mn < Pb, Nysa Szalona—Ni < Fe < Zn < Cd < Mn < Cu < Pb, Strzegomka—Ni < Cd < Fe < Zn < Cu < Pb < Mn. For all the samples studied, the values found in spring were much higher than in autumn, which indicates the great importance for research in that area. The levels of copper and iron were within the range of moderate values, lead and manganese reached very high and exceptionally high values, and the remaining metals were within the values described as significant. Bioaccumulation of metals determined relative to bottom sediments was highest in 2017 and lowest in 2018, while bioaccumulation relative to water was highest in 2018 and lowest in 2016. The four-year study found that the metal content in reed canary grass was mostly within the range of mean values presented in the literature from moderately polluted areas. Also, no significant deviation was found from levels that have been recorded for the same rivers for more than two decades.

## 1. Introduction

Natural levels of metals in the environment are disrupted by industrial, agricultural and municipal activities. Each type of activity often results in a different metal load to the air, soil, groundwater and surface water. Elevated levels of some metals are not the cause of significant changes in the environment and do not have a strong toxic effect on its components. However, there is a group of metals, the presence of which even at low concentrations can cause deterioration in the quality of the environment. All of the environmental components have an effect on human life, but some of them, such as waters being a source of drinking supply, should be under special supervision. Apart from groundwater reservoirs, this type of water includes surface waters—lakes and rivers as well as dam reservoirs built on them. They are supplied by river waters and accumulate material originating from catchment areas. The main rivers and their tributaries carry characteristic pollutants collected in the catchment. As a rule, in mountain catchments, in contrast to lowland catchments, the material is poorer in organic components and the components carried with the faster current have limited possibilities of being deposited in the form of bottom sediment. This can result in a reduced amount of available rooting sites for aquatic plants, and free-floating macrophytes are unlikely to find a place for themselves due to the rapid water flow. An additional factor depleting the number of plants in mountain riverbeds is their transformation by humans. This is associated with the ongoing regulation of rivers, which entails the concreting of their beds and banks, thus eliminating potential habitats for plant rooting, as well as reducing the species composition and abundance of organisms. As a consequence, the number of aquatic organisms treated as pollutant filterers carrying water from the catchment is reduced. Such activities are important from the point of view of using water for domestic purposes and as drinking water [1,2,3,4,5].

The amount of organic and mineral compounds carried with river waters is a result not only of natural but also anthropogenic activities. It may also be a further consequence of specific conditions for the release of metals from soil and sediments, which under favorable circumstances, e.g., excessive acidification, become more mobile and available for uptake by plants. The mobility of elements depends, among others, on the reaction of water. This is the case of metals, which become a threat to the state of water quality in a more strongly acidified or alkalized environment [6,7,8,9,10]. The presence of metals in aquatic plants is also related to their level in the atmospheric air. In the areas burdened by a stronger anthropogenic influence and subjected to natural activities of this character, their level in the air is higher, which is reflected in the chemical composition of soil and water, and further on in macrophytes. More than 90% of cadmium, copper, mercury, lead and zinc compounds in bottom sediments have their source in anthropogenic industrial pollution. Table 1 presents average the metal contents in particular components of the natural environment [11,12]. These elements belong to the group of trace, cyclic elements occurring most numerously in nature, constituting 99.9% of the mass of the Earth’s crust and playing a significant role in the environment. Their characteristic feature is the ability to react and repeatedly form the same compounds. In addition, manganese, iron and copper are defined as biophilic elements, as those which are part of living organisms and their circulation is largely related to the course of biological processes. As a rule, the natural circulation of elements takes place in a balanced way, but when anthropogenic activity becomes a stimulus, this order may be disturbed. It is visible in the process of accumulation of metals, among which there are groups more (Cu, Cd, Pb, Zn) or less susceptible to accumulation (Fe, Mn). All of these elements, in larger quantities, pose a threat to the environment, which can transform from a local impact into a threat of a wider scope. In the case of aquatic plants, susceptibility to bioaccumulation from water concerns mainly cadmium, lead, copper and zinc, and from bottom sediment—cadmium and zinc [12].

In the south-western part of Poland, where rivers and reservoirs are the main stores of drinking water, the study included three rivers: Nysa Szalona, Bystrzyca and Strzegomka, supplying the reservoirs Słup, Dobromierz and Lubachów, respectively. They differ in structure, but mainly in their location above sea level and character: lowland, submontane and mountainous.

Until now, no such comprehensive and long-term study of aquatic vegetation has been conducted within these reservoirs. Although some sections of rivers and reservoirs were included in the analysis, it was not a comprehensive study [12,13,14,15,16]. The authors studied water and bottom sediments simultaneously in this area [17,18]. All of these components would provide a holistic picture of the environment of these three catchments, including not only the dam reservoirs themselves, but the main rivers feeding them along with all tributaries. First of all, this study will make it possible to determine the quality of the environment from the natural point of view. Understanding the levels of metals in hydro-macrophytes is important from the point of view of plant health, but also their usefulness in cleaning the aquatic environment. Secondly, such comprehensive studies, as in this case, will make it possible to indicate potential threats to the quality of water which supply reservoirs intended for drinking water.

The aim of this study was to determine the possibility of accumulation of metals (Cu, Zn, Cd, Pb, Ni, Fe, Mn) in reed canary grass growing in rivers feeding the three reservoirs. Water from the reservoirs is taken as drinking water, so testing for water pollution and biotic elements is advisable. Reed canary grass is a plant that grows in all the beds of these rivers and may, in this situation, be used as an indicator plant.

## 2. Materials and Methods

### 2.1. Study Area

**Figure 1:**
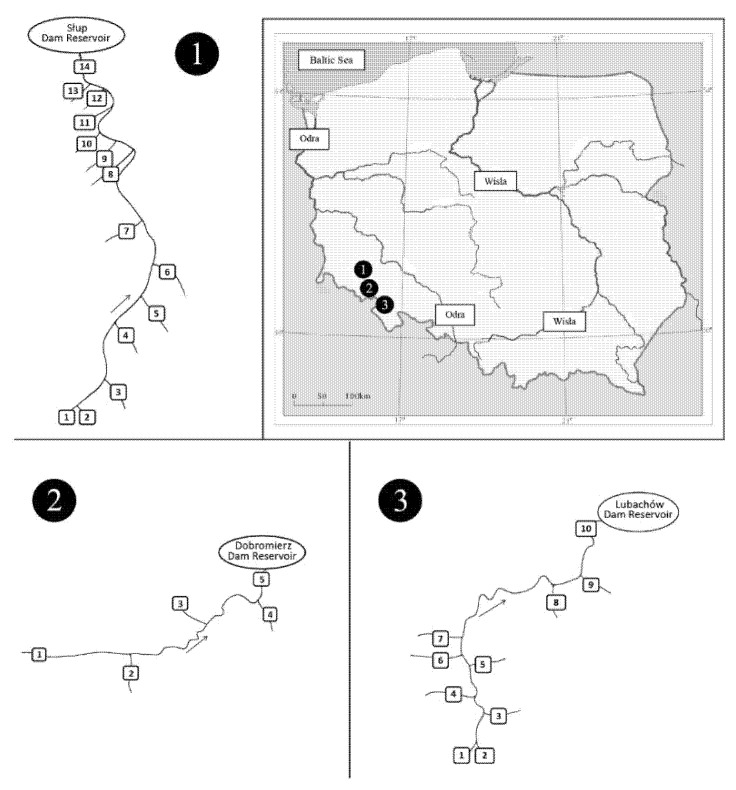
Location of the study area, 1—Słup reservoir—research sites on the Nysa Szalona River and its tributaries (Table 2), 2—Dobromierz reservoir—research sites on the Strzegomka River and its tributaries (Table 3), 3—Lubachów reservoir—research sites on the Bystrzyca River and its tributaries (Table 4).

The study covered the following areas in south-west Poland: N 50°38′10.1652″–N 51°4′31.7745″ and E 16°3′54.4715″–E 16°25′1.4097″ [19,20]. The study included three Lower Silesian rivers: Nysa Szalona, Strzegomka and Bystrzyca along with their tributaries from the sources to their mouths in the dam reservoirs Słup, Dobromierz and Lubachów (Figure 1; Table 2, Table 3 and Table 4).

The characteristics of the rivers under examination are presented in Table S1 [13,14,15,19,20].

### 2.2. Material

Reed canary grass (*Phalaris arundinacea* L.) was sampled twice a year at the beginning (May) and at the end of the growing season (October). The study was conducted from 2015 to 2018. Plants were collected from main rivers, and additionally from tributaries at a distance of 50 m before their mouths (Figure 1, Table 2, Table 3 and Table 4) [21,22,23].

Monocotyledonous emergent plants (helophytes) were sampled. Taxonomic position of the collected plants is as follows:

Domain: *Eucaryota*—eukaryotes
Kingdom: Archaeplastida (Plantae)—plants
Clade: *Spermatophyta*—spermatophytes
Class: *Liliopsida* (*Monocotyledones*)—monocotyledons
Order: Poales (Graminales)—Glumiflorae
Family: *Poeceae* (*Gramineae*)—grasses
Subfamily: *Pooideae*
Tribe: *Poeae*
Subtribe: Phalaridinae
Species: reed canary grass (*Phalaris arundinacea* L.)

Reed canary grass was harvested whole (root, stem, leaves, and inflorescence). Immediately at the test site after collection, plants were washed with river water. In the laboratory, they were dried at room temperature to an air-dry state and then cut, crushed, and homogenized.

For the determination of metals, 0.5 g of air-dry and homogenized sample was weighed in an HP-500 Teflon dish. Then, 10 cm^3^ of concentrated HNO_3_ (Sigma-Aldrich, Poznań, Poland) was added and the samples were left at room temperature for 24 h. After this time, the samples were placed in a Mars 5 microwave oven (CEM) and mineralized using a 3-stage mineralization. After cooling to room temperature, the mineralizates were transferred to test tubes and diluted with distilled water to 25 cm^3^ [24,25,26].

232 plant samples were collected and results are given in mg∙kg^−1^ dry weight.

The levels of lead, copper, nickel, zinc, cadmium, iron and manganese in aquatic plants were determined by flame atomic absorption spectrometry (FAAS) using a Spectra AA-110/220 from Varian [27].

Test results were verified using certified reference materials for aquatic plants—IAEA-336 International Atomic Energy Agency—Analytical Quality Control Services Austria and CRM 482—Commission of the European Communities, Community Bureau of Reference—BCR.

Accumulation of metals in aquatic plants was determined by:

metals bioaccumulation factor (BCF_B_) as a ratio of its content in aquatic plant (C_P_) to its concentration in bottom sediment (C_B_) [28]

$${\mathrm{BCF}}_{B}=\frac{C_{P}}{C_{B}}$$

metals bioaccumulation factor BCF_W_ as a ratio of its content in aquatic plant C_P_ to its concentration in water C_W_ [28]

$${\mathrm{BCF}}_{W}=\frac{C_{P}}{C_{W}}$$

The assessment of the state of plants contamination with metals was carried out using the metal pollution index (MPI) [29].

MPI = (Cf_1_ × Cf_2_…Cf*_n_*)^1/*n*^

where Cf_1_, Cf_2_…Cf*_n_*—concentration of first metal, second metal, *n*-th metal.

MPI values less than 2 indicate no impact on pollution degree, values 2–5—very low impact, 5–10 low impact, 10–20 medium impact, 20–50 high impact, 50–100 very high impact, above 100 the highest impact.

The metal contamination status of aquatic plants was also assessed using the metal enrichment factor (EF) [30].

EF = (Me/Al)_sample_/(Me/Al)_background_

Me—particular metal, Al—aluminum.

For individual metals, the following values were taken as background: Al—1708; Cd—1.03; Cu—4.95; Zn—40; Pb—1.45; Mn—115; Fe—210; Ni—9.51 [12].

EF values less than 2 indicate no or minimal enrichment of the plant with particular metals, range 2–5 as moderate, 5–20 as significant, 20–40 as very high, and above 40 as extremely high [30].

### 2.3. Statistical Analysis

The analysis of results was performed using Microsoft Office Excel 2019 and Statistica 13.0 (StatSoft Poland, Krakow, Poland). Calculations were performed using R version 3.6.0. The Shapiro-Wilk test was applied to check the normality of the distribution. Spearman correlations were used due to the distribution of samples. Spearman correlations were calculated in Statistica, and box and whisker plots were also created in this program. All statistically significant differences were calculated at *p* < 0.05. Because the data had a non-normal distribution, the Kruskal-Wallis test with post-hoc analysis was used. A PCA test using the r-groups statistic (RStudio Version 1.1.442-© 2022–2018, Rstudio, Inc., Boston, MA, USA) was used to visualize differences between groups. It was based on all data and presented the differences in the parameters of the studied plant depending on the year, season of study and river.

## 3. Results and discussion

### 3.1. Metals in Reed Canary Grass

The highest contents of Cu, Ni, Cd, Pb were recorded in reed canary grass sampled from the Nysa Szalona River (Table 5). Samples from the Bystrzyca River had the highest zinc levels, while samples from the Strzegomka River had the highest iron and manganese levels. The lowest values of Fe, Mn and Zn were recorded in plants obtained from the Nysa Szalona River, Pb and Cd from the Strzegomka River, and Cu and Ni from the Bystrzyca River. The series of metals were as follows: Nysa Szalona and Strzegomka: Cd < Ni < Pb < Cu < Zn < Mn < Fe, and Bystrzyca: Cd < Ni < Cu < Pb < Zn < Mn < Fe.

#### 3.1.1. Copper

Throughout the study cycle, the range of copper content in reed canary grass was from 2.33 mgCu·kg^−1^ to 396.55 mgCu·kg^−1^, with a range for mean values of 7.92 mgCu·kg^−1^–31.02 mgCu·kg^−1^ (Table 5). The bioaccumulation factor with respect to bottom sediments ranged from BCF_B_ = 0.0090 to BCF_B_ = 13.13, and with respect to water it was higher, at a level of BCF_W_ = 30.47–82,142.29 (Table 5).

**Figure 2:**
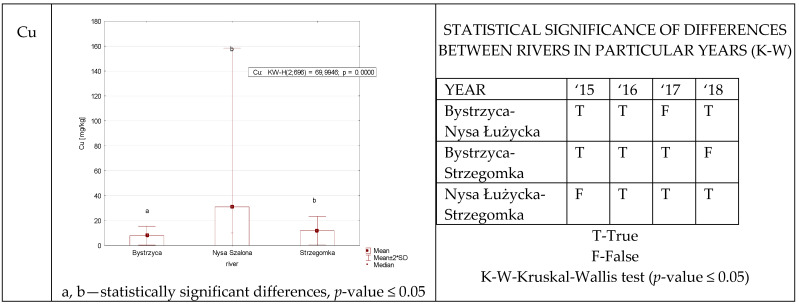
Cu concentration in plants from rivers across 29 sites depending on river (graph to the (**left**)) and by year 2015–2018 in the table to the (**right**). Statistically significant differences are marked in the graph with letters a, b and in the table these differences are shown by year between rivers—T—differences were present, F—no statistical difference (*p*-value ≤ 0.05).

Statistically significant differences in copper content of reed canary grass were recorded between all rivers in each year with minor exceptions (Figure 2). These exceptions indicate that each year was different, and therefore inferences about environmental quality should be made during long-term studies. The long-term data allowed the identification of the Nysa Szalona River as having statistically higher levels of this metal.

The content of copper in hydromacrophytes examined in the study compared to the results from previous years indicates a relatively unchanged level maintained for years in the case of the Strzegomka and Nysa Szalona Rivers (300–500 mgCu·kg^−1^) [16]. Plants collected from the Słup dam reservoir, into which the Nysa Szalona River flows, and from the Bystrzyca River supplying the reservoir in Lubachowo fall into a similar range [2,4]. Also in other reservoirs of flowing and standing waters of that region and not strongly influenced by economic activity, the level of copper in plants was similar to that presented in this study [31,32,33,34,35,36,37,38,39]. However, in the reservoirs of the same region, whose catchment areas are strongly exposed to greater pollution or even contamination (copper mine), an increase in the level of this metal in aquatic plants can be observed [40].

Studies conducted in other parts of Poland and the world in moderately polluted areas show that although copper levels in plants were quite variable, they were similar to those recorded for the studied rivers [5,36,38,41,42,43,44,45,46,47,48,49,50,51].

#### 3.1.2. Cadmium

Cadmium content in reed canary grass ranged from 0.0400 mgCd·kg^−1^ to 10.89 mgCd·kg^−1^ (Table 5). Mean values ranged from 0.8969 mgCd·kg^−1^ to 1.65 mgCd·kg^−1^. Cadmium bioaccumulation factor with respect to sediment (BCF_B_ = 0.0209–41.07) was lower than with respect to water (BCF_W_ = 5.35–18,155.50) (Table 5). In studies on the Strzegomka, Nysa Szalona and Bystrzyca Rivers conducted in previous years, the cadmium content in plants was similar to the results obtained in this study, with levels reaching a maximum of 8.80 mgCd·kg^−1^ [2,4,5,16,31,32,35,37,38,39,40,41,42,43,45,46,47,48,49,51,52]. This indicates a persistently stable level of cadmium in the environment and relatively unchanged water chemistry conditions. Higher concentrations were recorded in submerged plants or those growing in intensively polluted reservoirs such as post-mining pits, for example [33,34,36,53].

**Figure 3:**
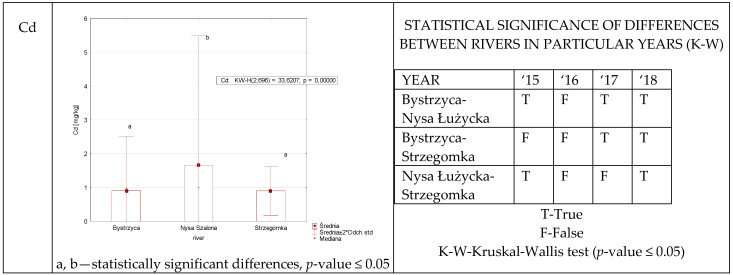
Cd concentration in plants from rivers across 29 sites depending on river (graph to the (**left**)) and by year 2015–2018 in the table to the (**right**). Statistically significant differences are marked in the graph with letters a, b and in the table these differences are shown by year between rivers—T—differences were present, F—no statistical difference (*p*-value ≤ 0.05).

Statistically significant differences in cadmium levels in aquatic plants were found between all rivers only in the last year of the study (Figure 3). In contrast, no such differences were recorded between any rivers in 2016. The multiannual data obtained indicated that both the Bystrzyca and Strzegomka have statistically the lowest cadmium content compared to the Nysa Szalona.

#### 3.1.3. Nickel

Nickel levels in all plant samples collected ranged from 0.1625 mgNi·kg^−1^ to 31.67 mgNi·kg^−1^ (Table 5). The mean values ranged between 5.12 mgNi·kg^−1^ and 6.76 mgNi·kg^−1^. The bioaccumulation of nickel determined with respect to bottom sediments was BCFB = 0.0041–1.57, and a higher level was reached with respect to water, BCF_W_ = 0.7329–13,014.50 (Table 5).

The Bystrzyca River and the Słup Dam reservoir, into which the Nysa Szalona flows, were characterized in previous years by similar nickel levels to those presented in this study [2,4]. This indicates a fairly stable level of nickel in the studied environment, although in studies on the Nysa Szalona and Strzegomka carried out in earlier years the level of nickel was higher [34]. Higher levels of nickel were also found in submerged plants and those growing in artificial reservoirs, but also in mountainous and lowland areas subjected to anthropopression [36,40,50].

In general, in most of the studies presented, the range of nickel content in aquatic plants is similar to that recorded in this study and reaches a maximum threshold at 24.67 mgNi·kg^−1^ [32,33,37,38,39,41,43,45,46,49,51,52,54].

**Figure 4:**
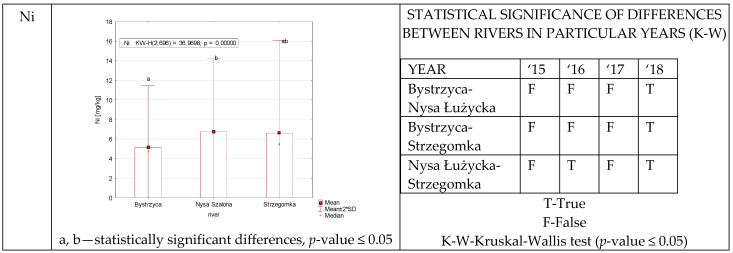
Ni concentration in plants from rivers across 29 sites depending on river (graph to the (**left**)) and by year 2015–2018 in the table to the (**right**). Statistically significant differences are marked in the graph with letters a, b and in the table these differences are shown by year between rivers—T—differences were present, F—no statistical difference (*p*-value ≤ 0.05).

Similar to cadmium, statistically significant differences in nickel levels between plants from different rivers were found in 2018 (Figure 4). In the remaining years, such differences were not observed, although in sum, without division into years, statistical differences were observed between the Bystrzyca, and the Nysa Szalona.

#### 3.1.4. Lead

Lead levels in plants ranged from 1.63 mgPb·kg^−1^ to 87.36 mgPb·kg^−1^, with averages oscillating between 8.74 mgPb·kg^−1^ and 22.26 mgPb·kg^−1^ (Table 5). Bioaccumulation with respect to water was BCF_W_ = 6.03–219,934.00, and much less with respect to sediment BCF_B_ = 0.0013–3.79 (Table 5).

Similar values to the present results were noted for the same rivers in previous years, which as for nickel, copper and cadmium confirms relatively stable conditions present in the rivers for many years [2,4,16]. However, higher values were found in plants from mountainous regions, but also from highly industrialized and urbanized areas [34,38,40,48,53].

**Figure 5:**
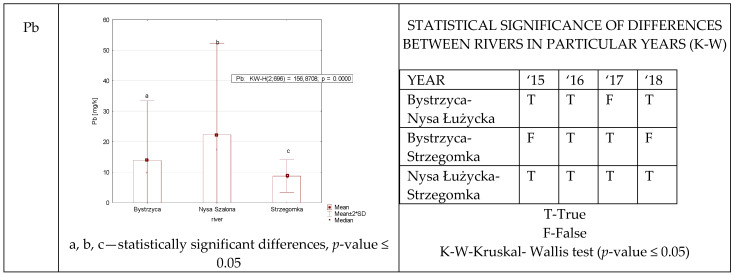
Pb concentration in plants from rivers across 29 sites depending on river (graph to the (**left**)) and by year 2015–2018 in the table to the (**right**). Statistically significant differences are marked in the graph with letters a, b and in the table these differences are shown by year between rivers—T—differences were present, F—no statistical difference (*p*-value ≤ 0.05).

Only in 2016, statistically significant differences in lead content in reed canary grass were found (Figure 5) between all studied rivers. In 2015 and 2018 such differences were found only between the Bystrzyca and the Nysa Szalona, and between the Nysa Szalona and the Strzegomka. The multiannual data collected allowed for the observation of the lowest amount of lead in the Strzegomka and the highest in the Nysa Szalona.

#### 3.1.5. Zinc

The amount of zinc in reed canary grass ranged from 5.96 mgZn·kg^−1^ to 206.54 mgZn·kg^−1^, with a range for mean values of 44.54–4742.30 mgZn·kg^−1^ (Table 5). Zinc accumulation in plants relative to sediment (BCF_B_ = 0.0099–3.46) was lower than relative to water (BCF_W_ = 13.84–70,158.80).

The level of zinc, similarly to the above-mentioned metals, in previous studies carried out in the area of the Słup reservoir into which the Nysa Szalona flows, was within a similar range as at present (8.00–80.10 mgZn·kg^−1^) [2]. However, for the Nysa Szalona, Strzegomka and Bystrzyca Rivers studied separately, the values found were higher (up to 700 mgZn·kg^−1^) [4,16]. Higher values were also found in the submontane regions of Lower Silesia and other rivers in southern Poland, as well as in the western border areas of Germany [32,33,34,38]. Higher values were also found in more industrialized regions [40,49]. Generally, in the majority of studies covering the European area and other parts of the world, the level of copper is similar to that recorded in this study [2,5,31,37,39,41,42,43,44,45,46,47,48,50,51,52,54,55].

**Figure 6:**
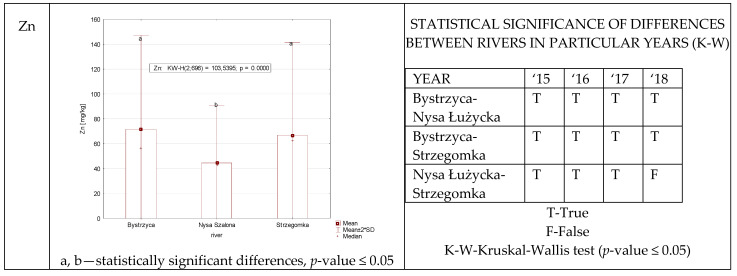
Zn concentration in plants from rivers across 29 sites depending on river (graph to the (**left**)) and by year 2015–2018 in the table to the (**right**). Statistically significant differences are marked in the graph with letters a, b and in the table these differences are shown by year between rivers—T—differences were present, F—no statistical difference (*p*-value ≤ 0.05).

Statistically significant differences in zinc content were recorded with one exception (year 2018, Nysa Szalona and Strzegomka) between all rivers (Figure 6). All the obtained data allowed this study to single out the Nysa Szalona River as the watercourse with the statistically lowest level of zinc.

#### 3.1.6. Iron and Manganese

Iron levels in the aquatic plant samples ranged from 2.96 mgFe·kg^−1^ to 6597.40 mgFe·kg^−1^, with mean values of 461.31–1257.50 mgFe·kg^−1^ (Table 5). Bioaccumulation of iron calculated relative to its level in water was BCF_W_ = 1.23–16,874.94, and relative to bottom sediments it was lower, BCF_B_ = 0.0001–6.92 (Table 5).

The manganese content in reed canary grass ranged from 12.03 mgMn·kg^−1^ to 3955.12 mgMn·kg^−1^ (Table 5). Mean values ranged from 186.45 mgMn·kg^−1^ to 213.58 mgMn·kg^−1^. Manganese bioaccumulation with respect to water was BCF_W_ = 22.93–21,392.88, and with respect to bottom sediments it was BCF_B_ = 0.0681–35.58 (Table 5).

The level of iron and manganese in plants studied in the Bystrzyca River remained within a similar range as in previous years [4]. Also in other rivers in this region of Poland the iron content in hydromacrophytes was similar [31,32,33,35,39,54]. Apart from that area, the recorded metal contents in aquatic plants were similar in other reservoirs [49,52,56].

However, significantly higher amounts were found in plants from the Nysa Szalona and Strzegomka Rivers studied several years earlier and in industrial areas, which indicates the anthropogenic origin of these metals [16,32,34,37,40,50].

**Figure 7:**
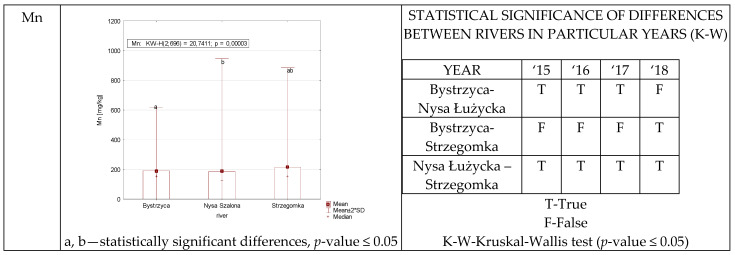
Mn concentration in plants from rivers across 29 sites depending on river (graph to the (**left**)) and by year 2015–2018 in the table to the (**right**). Statistically significant differences are marked in the graph with letters a, b and in the table these differences are shown by year between rivers—T—differences were present, F—no statistical difference (*p*-value ≤ 0.05).

Statistically significant differences in manganese content were noted between the Nysa Szalona and Strzegomka in each year of study (Figure 7). Statistically significant differences were recorded between the Bystrzyca and the Nysa Szalona. The exception was the year 2018, where these differences were not found, exactly the opposite statistical relationship was observed between the Bystrzyca and Strzegomka Rivers.

**Figure 8:**
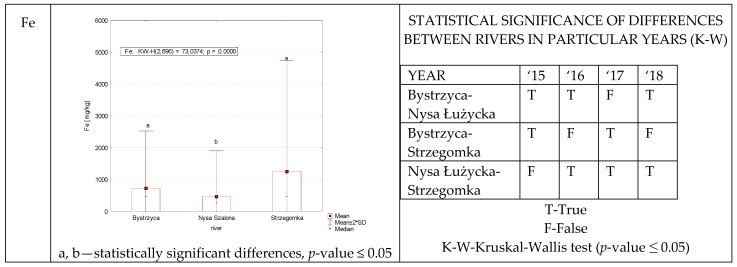
Fe concentration in plants from rivers across 29 sites depending on river (graph to the (**left**)) and by year 2015–2018 in the table to the (**right**). Statistically significant differences are marked in the graph with letters a, b and in the table these differences are shown by year between rivers—T—differences were present, F—no statistical difference (*p*-value ≤ 0.05).

In the case of iron, statistically significant differences in its content in the studied samples recorded between the three rivers do not allow to determine the regularity (Figure 8). The four-year study made it possible to show the Nysa Szalona as the river with the statistically lowest level of iron.

**Figure 9:**
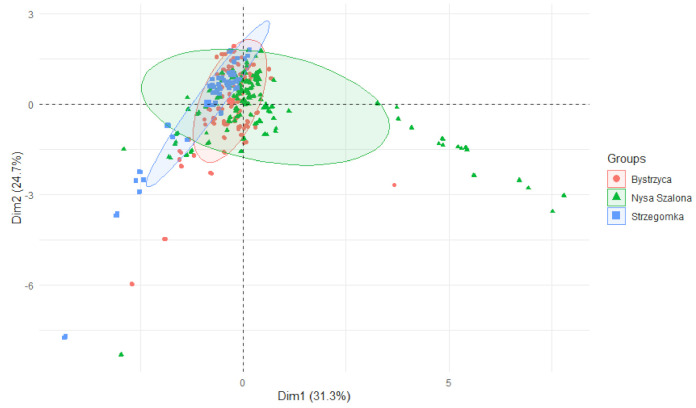
PCA plot 2D showing clustering of metals concentration in plants from rivers across 29 sites and 4 years (2015–2018) grouping factor—river.

The differences between the rivers may be noted using PCA plot. Nysa Szalona and Strzegomka were the most different in respect of the examined parameters, and Bystrzyca had parameters intermediate between the other rivers (Figure 9).

**Figure 10:**
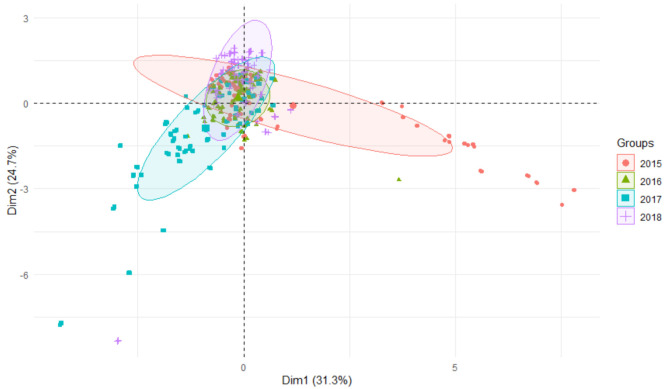
PCA plot 2D showing clustering of metals concentration in plants from rivers across 29 sites and 4 years (2015–2018) grouping factor—year.

During the four-year study cycle, for all metals studied, the lowest values were recorded in the first (2015) and last (2018) year of the study, and the highest in 2017 (Table 5). Bioaccumulation of metals determined relative to bottom sediments was highest in 2017 and lowest in 2018, while bioaccumulation relative to water reached the highest values in 2018 and lowest in 2016. Using the PCA plot, differences between years can be seen, with the most varied results in 2015 and the least varied results in 2016 (Figure 10).

### 3.2. Comparison of Metals Content—Source and Mouth of the River

Comparison of metal levels within the extreme sites on the main rivers shows that, for the most part, the estuaries of the rivers to the reservoirs in all study years had higher contents of the metals studied than the site below the springs (Table 6). This indicates enrichment with metal compounds of the main rivers by successively escaping tributaries. A similar relationship was found for the accumulation coefficient in relation to sediment. On the other hand, for the coefficient calculated in relation to metal concentration in water, sometimes higher values occurred at the site downstream of the springs.

Metal contents also varied with the seasons. In the Strzegomka River at both extreme sites, higher amounts of Cu, Ni, Cd, Pb and Mn were recorded in autumn, and Zn and Fe in spring (Table 7). The Bystrzyca River had higher values for all metals at the reservoir outlet in spring (Table 8). Below the springs, there was a differentiation: in spring, higher levels of cadmium, lead and zinc were recorded, whereas the remaining metals predominated in autumn. On the other hand, in the Nysa Szalona River at both sites, copper and cadmium were more abundant in the spring, and higher amounts for the other metals were recorded in the autumn (Table 9).

**Figure 11:**
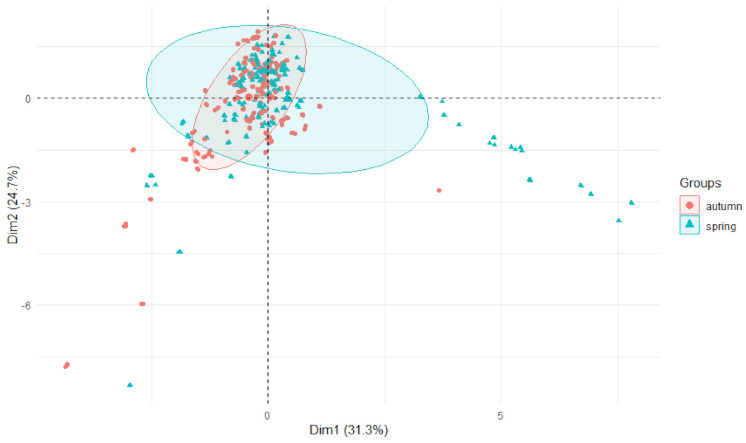
PCA plot 2D showing clustering of metals concentration in plants from rivers across 29 sites and 4 years (2015–2018) grouping factor—season.

Furthermore, differences between the spring and autumn seasons are noticeable. Spring was the most differentiated season with respect to the studied parameters (Figure 11).

### 3.3. Metals in Tributaries of Major Rivers

The analysis of metal contents in plants taken from successive tributaries of the three main rivers shows that in the Strzegomka River, the level of Cu, Ni, Cd, Fe, Mn increased with the river course and in the middle part the content of these metals was the highest. For lead, no major changes were observed in the whole river, and the range of values was 7.12 to 9.58 mgPb·kg^−1^ (Table 7). For zinc, values decreased with the river course from 89.90 mgZn·kg^−1^ to 63.46 mgZn·kg^−1^ in spring, and from 56.49 mgZn·kg^−1^ to 47.89 mgZn·kg^−1^ in autumn.

In the Bystrzyca River, copper, nickel, and cadmium levels increased downstream (Table 8). In the case of zinc, the upper and lowland tributaries contributed higher amounts than the midstream rivers. For iron and manganese, decreasing values were observed with the direction of water flow in the river.

In the case of the Nysa Szalona River, the amount of copper and lead in plant samples increased steadily with successive tributaries (Table 9). It was also observed that the midstream rivers contributed higher amounts of cadmium, zinc and manganese, and lower amounts of iron. Nickel remained at an even level in all tributaries.

**Figure 12:**
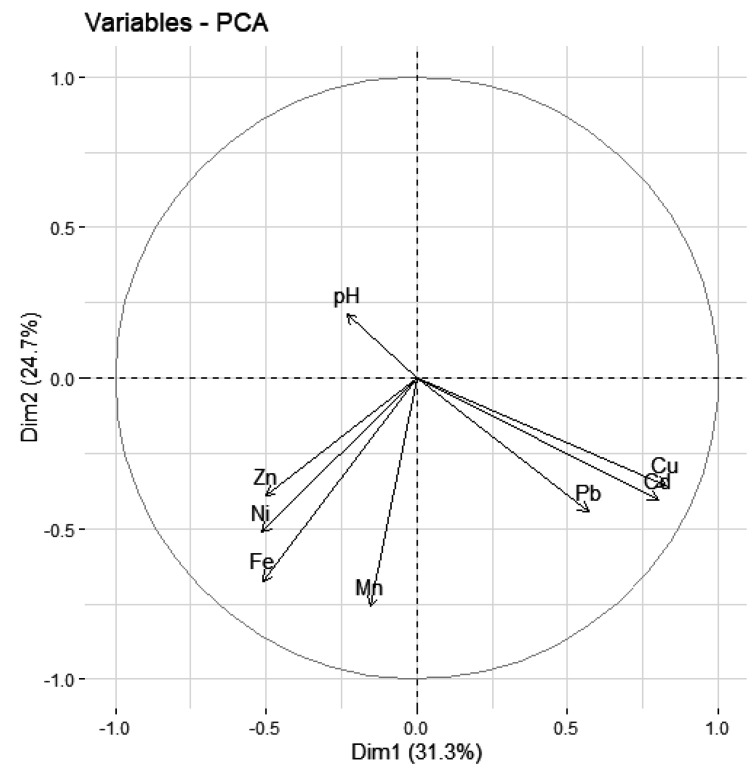
Ordination of the 29 study sites by PCCA based on concentrations of elements in plants and water (pH) in all investigated rivers.

Two groups of elements clearly emerge from the study. The first one includes Pb, Cu and Cd, and they turned out to be statistically highly correlated (Figure 12). The second group of metals includes Zn, Ni, Fe, Mn which are a group of elements correlated with each other. More detailed values of correlations are given in Table 10.

The investigated river ecosystem covers a considerable area of the south-western part of Poland. In this area, besides groundwater, rivers as surface waters are the main source of drinking water supply for the population. The content of metals in plants growing in the riverbeds which supply the reservoirs from which drinking water is obtained suggests that they do not pose a threat to human life. The level of metals in hydromacrophytes is within the average limits for these types of waters in this climatic zone. This is confirmed by the results of the studies by the authors cited above [2,4,17,18,35,39,54]. Also, the concentrations of metals in river water recorded throughout the four-year study cycle are low enough to meet the criteria for water extracted by water production plants for water production [57,58]. If the natural environment continues to be so stable, and there are no changes in the reaction of water and bottom sediments, it can be assumed that the current level of metals in water and plants will not pose a threat to human health in this area in the future

### 3.4. Metal Pollution Index (MPI) and Enrichment Factor (EF)

The metal pollution index (MPI) of aquatic plants was used to compare the metal content of samples from different sites [29]. The general pattern of MPI in aquatic plants was arranged in a series B < S < NS, and the same pattern was found for water, however a slightly different order occurred for sediments: S < B < NS (Table 11) [17,18]. During the four-year cycle of the study, higher MPI values were recorded in 2016 and 2018 than in 2015 and 2017 (Table 12).

These values classify the studied material at high pollution degree in all rivers. Against this background, previously published data for sediment reached the highest and very high grades [18]. In the case of the MPI for water, no effect of pollution was found [17]. With this comparison, it can be seen that it is only when metal levels in sediments and plants are studied that the accumulation of metals in the environment becomes apparent. Hence, conducting a comprehensive study is justified. In the comparison of the two extreme sites: source–mouth, the predominance of higher values at the reservoir mouth in the first three years of the study is evident, indicating the origin of these metals from the catchment. On the other hand, in the last year (2018) higher MPI values were found at the sources in the Nysa Szalona and Bystrzyca Rivers (Table 12).

The enrichment factor (EF) calculated for all plant samples reached the lowest values for nickel and the highest values for lead and manganese (Table 13, Table 14 and Table 15). The series of increasing values were as follows: Bystrzyca—Ni < Cd < Fe < Cu < Zn < Mn < Pb, Nysa Szalona—Ni < Fe < Zn < Cd < Mn < Cu < Pb, Strzegomka—Ni < Cd < Fe < Zn < Cu < Pb < Mn. For all studied plants in each of the three rivers, the values were definitely higher in spring than in autumn.

In the Bystrzyca River, higher values of the enrichment factor EF were found at the site immediately upstream of the reservoir and in the Nysa Szalona River at the site downstream of the springs. In the Strzegomka River, a differentiation was observed: higher values for Cu, Ni, Fe and Mn were recorded downstream of the source, while the remaining metals were observed at the upstream site.

For most samples, Cu and Fe are within the range of moderate values. Lead and manganese reach very high and extremely high values. The remaining metals fall in the values described as significant. This assessment suggests that the amount of metals absorbed by reed canary grass varies. While for the time being there is no concern about the levels of copper and iron, for the rest of the metals a more frequent, regular study should be carried out.

## 4. Conclusions

A four-year study of reed canary grass in rivers of southwestern Poland ranked metal levels as follows: Nysa Szalona and Strzegomka: Cd < Ni < Pb < Cu < Zn < Mn < Fe, and Bystrzyca: Cd < Ni < Cu < Pb < Zn < Mn < Fe. Throughout the study period, the lowest values of metals in plants were recorded in 2015 and 2018, and the highest in 2017.

The general picture of MPI in aquatic plants is arranged in the series Bystrzyca < Strzegomka < Nysa Szalona. These values classify the studied material at a high level of pollution in all rivers. In the comparison of two extreme sites, i.e., source–mouth, higher values are found at the mouth of the reservoir, which suggests that metals move with the water current and accumulate more with the direction of the river flow, which is most likely a consequence of the influence of the catchment area as the source of metals.

The series of enrichment factor (EF) values were as follows: Bystrzyca—Ni < Cd < Fe < Cu < Zn < Mn < Pb, Nysa Szalona—Ni < Fe < Zn < Cd < Mn < Cu < Pb, Strzegomka—Ni < Cd < Fe < Zn < Cu < Pb < Mn. For all the samples studied, the values found in spring were much higher than in autumn, which would indicate the great importance of conducting research in that area.

Bioaccumulation of metals determined relative to bottom sediments was highest in 2017 and lowest in 2018, while bioaccumulation relative to water was highest in 2018 and lowest in 2016.

The four-year study found that the metal content in reed canary grass was mostly within the range of the mean values presented in literature data from moderately polluted areas. Also, no significant deviation was found from levels that have been recorded for the same rivers for more than two decades.

## Figures and Tables

**Table 1:** Ranges of metal content in environmental components [11,12].

Metal/Unit	Air	Waters	Soils	Plants
	ng∙m^−3^	mg∙dm^−3^	mg∙kg^−1^	mg∙kg^−1^
Cu	0.03–4900.00	0.001–0.020	3.00–25.00	5.00–30.00
Cd	0.003–0.60	0.00001–0.00002	0.0002–0.60	0.05–0.20
Ni	0.10–1.00	0.0001–0.0075	5.00–22.00	0.10–5.00
Pb	0.50–10.00	0.0002–0.0003	25.00–40.00	0.10–5.00
Zn	0.002–0.05	1.00–110.00	10.00–220.00	10.00–70.00
Fe	0.50–6000.00	0.01–1.40	8000.00–18,000.00	50.00–200.00
Mn	0.02–900.00	0.02–0.06	100.00–1300.00	70.00–500.00

**Table 2:** Research sites—the Nysa Szalona River and its tributaries above the Słup dam reservoir [19].

No	Site—Geographical Coordinates	Surface Water Types, Water Categories—Type Code SWB * Status
1	The Nysa Szalona River below the springs in Domanów—N 50°51′38.8261″ E 16°3′54.4715″	Upland silicate stream with coarse-grained substrate—western 4 natural
2	Kocik—N 50°52′15.4891″ E 16°4′5.9042″	
3	Ochodnik—E 16°5′59.7672″	
4	Sadówka—N 50°55′58.609″ E 16°10′11.3627″	
5	Czyściel—N 50°57′49.4252″ E 16°13′57.6982″	
6	Radynia—N 50°58′56.648″ E 16°14′13.9202″	
7	Nysa Mała—N 51°0′10.455″ E 16°12′26.0825″	Upland carbonate stream with coarse-grained substrate 7 natural
8	Puszówka—N 51°2′30.3945″ E 16°11′39.425″	
9	Jawornik—N 51°2′57.6884″ E 16°10′52.4584″	
10	Księginka—N 51°3′17.4033″ E 16°10′11.2082″	
11	Starucha—N 51°4′31.7745″ E 16°9′17.7528″	Upland silicate stream with fine-grained substrate—western 5 natural
12	Rowiec—N 51°4′22.844″ E 16°8′27.5419″	
13	Męcinka—N 51°4′29.2507″ E 16°7′28.5247″	
14	Nysa Szalona mouth to the Słup reservoir— N 51°4′29.2507″ E 16°7′28.5247″	Small upland silicate river—western 8 artificial watercourse

* SWB—surface water body.

**Table 3:** Research sites—the Strzegomka River and its tributaries above the Dobromierz dam reservoir [19].

No	Site—Geographical Coordinates	Surface Water Types, Water Categories—Type Code SWB * Status
1	The Strzegomka River below the springs in Nowe Bogaczowice—N 50°50′14.5978″ E 16°7′49.845″	Upland silicate stream with coarse-grained substrate—western 4 artificial
2	Polska Woda—N 50°52′48.0601″ E 16°11′56.4194″	
3	Sikorka—N 50°51′47.2613″ E 16°13′21.3918″	
4	Czyżynka—N 50°52′15.8303″ E 16°14′29.8332″	
5	Strzegomka mouth to the Dobromierz reservoir—N 50°53′11.1994″ E 16°13′58.4707″	Upland silicate stream with coarse-grained substrate—western 4 artificial

* SWB—surface water body.

**Table 4:** Research sites—the Bystrzyca River and its tributaries above the Lubachów dam reservoir [19].

No	Site—Geographical Coordinates	Surface Water Types, Water Categories—Type Code SWB * Status
1	The Bystrzyca River below the springs in Wrześnik— N 50°38′10.1652″ E 16°24′5.7915″	Upland silicate stream with coarse-grained substrate—western 4 artificial
2	Złoty Potok—N 50°38′29.3697″ E 16°24′41.0163″	
3	Kłobia—N 50°40′9.374″ E 16°23′27.0131″	
4	Otłuczyna—N 50°40′36.2015″ E 16°22′46.8444″	
5	Potok Marcowy Duży—N 50°41′5.2762″ E 16°22′32.3218″	
6	Złota Woda—N 50°41′4.2973″ E 16°22′11.0015″	
7	Rybna—N 50°41′49.8085″ E 16°21′58.1784″	
8	Jaworzynik—N 50°43′25.8799″ E 16°23′56.5218″	
9	Walimianka—N 50°43′49.9381″ E 16°24′15.0612″	
10	Bystrzyca mouth to the Lubachów reservoir— N 50°45′5.8065″ E 16°25′1.4097″	Upland silicate stream with coarse-grained substrate—western 4 artificial

* SWB—surface water body.

**Table 5:** Metal content (mg·kg^−1^) of reed canary grass in three main rivers and their tributaries over a four-year study cycle.

Metal	River	Index	2015	2016	2017	2018
				Min–Max $\bar{x}$ ± SD		
Cu	NS	P	3.69–396.55 92.79 ± 105.07	4.96–19.74 9.40 ± 2.99	2.33–20.98 10.85 ± 4.90	3.44–27.89 11.04 ± 5.80
			31.02 ± 63.63			
		BCF_B_	0.0090–13.13 2.92 ± 3.63	0.0190–0.5860 0.1550 ± 0.15	0.0596–2.12 0.8538 ± 0.58	0.1037–1.58 0.5280 ± 0.34
			1.11 ± 2.14			
		BCF_W_	923.00–82,142.29 19,842.46 ± 23,950.61	56.26–232.98 134.06 ± 46.13	664.66–5878.06 2637.96 ± 1622.71	40.60–2045.71 319.32 ± 373.18
			5733.45 ± 14,540.62			
	B	P	3.36–15.89 8.15 ± 2.95	3.96–9.00 6.66 ± 1.30	2.89–23.78 10.09 ± 5.83	3.45–13.47 6.76 ± 2.26
			7.92 ± 3.78			
		BCF_B_	0.0154–2.77 0.4269 ± 0.59	0.0107–1.14 0.3214 ± 0.29	0.2479–2.97 0.8282 ± 0.66	0.0982–1.63 0.6413 ± 0.42
			0.5545 ± 0.55			
		BCF_W_	437.38–15,894.30 3916.17 ± 2871.31	919.94–5736.77 1896.46 ± 1042.13	484.54–4634.65 2360.48 ± 1148.15	32.31–247.52 92.79 ± 47.97
			2066.48 ± 2126.21			
	S	P	9.41–13.98 11.74 ± 1.31	8.07–29.89 16.47 ± 7.26	9.34–22.68 13.59 ± 3.55	3.02–7.93 5.75 ± 1.58
			11.89 ± 5.72			
		BCF_B_	0.6326–1.99 1.24 ± 0.38	0.6109–4.25 1.57 ± 1.00	0.9014–2.24 1.32 ± 0.37	0.2281–1.11 0.5561 ± 0.26
			1.17 ± 0.69			
		BCF_W_	1300.77–5589.87 2385.15 ± 1255.77	1832.68–4519.43 3133.91 ± 904.62	1355.33–9448.13 3102.78 ± 2409.48	30.47–157.37 86.93 ± 44.08
			2177.04 ± 1896.46			
Cd	NS	P	0.5964–9.87 3.64 ± 3.05	0.5632–1.96 0.99 ± 0.26	0.4512–1.83 1.07 ± 0.29	0.5612–1.13 0.8932 ± 0.16
			1.65 ± 1.92			
		BCF_B_	0.1021–21.78 4.95 ± 5.92	0.1091–7.85 1.02 ± 1.45	0.4114–2.4820 1.11 ± 0.44	0.4733–41.07 1.21 ± 4.37
			2.09 ± 4.10			
		BCF_W_	504.68–10,256.00 3665.21 ± 2645.85	265.50–9652.00 1602.14 ± 1800.53	760.50–15,345.00 3877.44 ± 3409.34	13.96–1447.43 70.79 ± 242.36
			2303.89 ± 2816.61			
	B	P	0.22–1.24 0.76 ± 0.30	0.6312–10.89 1.09 ± 1.28	0.8712–2.09 1.31 ± 0.31	0.04–2.34 0.45 ± 0.57
			0.9046 ± 0.80			
		BCF_B_	0.2575–4.09 0.9804 ± 0.86	0.2020–10.86 1.60 ± 1.70	0.6409–3.24 1.67 ± 0.74	0.0209–2.20 0.50 ± 0.57
			1.1860 ± 1.17			
		BCF_W_	35.85–12,389.00 2785.70 ± 2956.48	1308.33–18,155.50 4291.59 ± 3190.04	1244.57–16,091.00 6401.30 ± 5395.53	71.40–12,385.00 1626.52 ± 2633.81
			3776.28 ± 4114.76			
	S	P	0.74–1.16 0.92 ± 0.12	0.7412–1.25 0.93 ± 0.13	0.1111–2.08 1.05 ± 0.52	0.1111–1.45 0.69 ± 0.39
			0.8969 ± 0.36			
		BCF_B_	0.8831–1.71 1.25 ± 0.25	0.8567–2.21 1.44 ± 0.37	0.1297–2.99 1.59 ± 0.92	0.1297–1.68 0.7913 ± 0.45
			1.27 ± 0.63			
		BCF_W_	534.06–8644.00 1909.34 ± 2268.10	828.58–7532.00 2320.29 ± 1560.09	185.17–17,076.00 2870.36 ± 4765.91	5.35–44.55 18.16 ± 9.89
			1779.54 ± 2953.55			
Ni	NS	P	0.2654–15.66 5.46 ± 5.33	3.25–9.35 6.46 ± 1.62	3.34–18.44 7.98 ± 3.22	1.72–14.84 7.15 ± 3.18
			6.76 ± 3.71			
		BCF_B_	0.0061–0.0787 0.0233 ± 0.01	0.0059–0.3128 0.1006 ± 0.10	0.0663–0.8549 0.2922 ± 0.17	0.0212–0.5079 0.1891 ± 0.11
			0.1513 ± 0.15			
		BCF_W_	63.16–13,014.50 4904.16 ± 4274.31	21.91–200.52 86.29 ± 45.55	146.06–5273.90 1740.10 ± 868.51	12.74–943.87 100.54 ± 185.67
			1707.77 ± 2936.45			
	B	P	1.63–8.27 4.38 ± 1.95	3.34–9.46 6.22 ± 1.60	3.73–13.98 8.09 ± 3.11	0.1625–4.35 1.81 ± 1.54
			5.12 ± 3.16			
		BCF_B_	0.0061–1.12 0.2746 ± 0.31	0.0187–0.9039 0.3287 ± 0.27	0.1189–1.03 0.4492 ± 0.23	0.0041–0.3889 0.1081 ± 0.10
			0.2900 ± 0.27			
		BCF_W_	1088.07–8872.25 4070.95 ± 2000.95	2224.60–7880.50 3777.76 ± 1349.49	199.35–3233.07 1870.45 ± 765.77	0.7329–42.14 16.66 ± 14.51
			2408.95 ± 2049.19			
	S	P	4.42–9.07 6.11 ± 1.26	2.95–8.49 5.29 ± 1.63	4.37–31.67 10.85 ± 7.58	2.88–6.91 4.16 ± 1.06
			6.60 ± 4.71			
		BCF_B_	0.2072–0.3550 0.2884 ± 0.05	0.1305–0.3908 0.2518 ± 0.08	0.2241–1.57 0.5080 ± 0.37	0.1356–0.3515 0.1990 ± 0.06
			0.3111 ± 0.23			
		BCF_W_	28.32–2108.77 700.64 ± 709.76	717.68–2178.60 1311.65 ± 364.22	30.77–7256.98 1648.45 ± 2182.31	15.35–51.48 29.99 ± 8.54
			922.69 ± 1315.60			
Pb	NS	P	13.00–87.36 36.82 ± 18.85	10.43–29.88 16.65 ± 4.04	6.02–38.65 16.82 ± 7.75	1.73–47.55 18.74 ± 13.53
			22.26 ± 15.00			
		BCF_B_	0.0513–2.76 0.6813 ± 0.75	0.0185–0.8832 0.3040 ± 0.26	0.1039–3.79 0.9058 ± 0.89	0.1027–1.86 0.7196 ± 0.45
			0.6527 ± 0.6734			
		BCF_W_	245.43–56,963.20 15,757.36 ± 15,294.00	3257.25–8316.06 5692.71 ± 1346.02	2153.48–48,141.50 14,263.33 ± 9914.17	6.03–8807.83 362.88 ± 1528.85
			9019.07 ± 11,127.08			
	B	P	2.41–16.07 5.91 ± 3.24	17.43–42.85 25.71 ± 6.98	8.45–28.95 17.57 ± 5.60	1.63–11.51 6.29 ± 2.84
			13.87 ± 9.72			
		BCF_B_	0.0013–0.2969 0.0645 ± 0.07	0.0099–0.8208 0.2841 ± 0.24	0.0736–0.5899 0.3056 ± 0.14	0.0102–0.1984 0.0988 ± 0.05
			0.1880 ± 0.18			
		BCF_W_	471.17–38,613.00 5355.71 ± 7137.87	7812.33–24,963.20 18,932.01 ± 3872.30	2224.36–219,934.00 21,749.61 ± 39,393.70	549.78–7290.42 2811.04 ± 1588.59
			12,212.09 ± 21,747.66			
	S	P	4.37–12.05 7.64 ± 2.08	5.13–14.65 9.02 ± 2.66	5.63–15.98 10.26 ± 3.03	3.91–10.94 8.02 ± 1.81
			8.74 ± 2.64			
		BCF_B_	0.0457–0.3607 0.1495 ± 0.08	0.0515–0.2591 0.1313 ± 0.06	0.0843–0.4159 0.1948 ± 0.1056	0.0618–0.2174 0.1121 ± 0.04
			0.1512 ± 0.08			
		BCF_W_	1213.83–11,348.50 3983.48 ± 2733.79	2052.88–12,733.14 5682.49 ± 3019.55	2157.64–25,128.60 6934.89 ± 6275.36	11.34–78.23 34.89 ± 14.68
			4158.93 ± 4556.24			
Zn	NS	P	5.96–66.87 33.04 ± 18.70	22.03–70.01 44.32 ± 13.06	25.68–128.72 64.89 ± 28.20	15.09–69.74 35.91 ± 13.79
			44.54 ± 23.06			
		BCF_B_	0.0099–0.9874 0.1400 ± 0.18	0.0142–0.9100 0.2511 ± 0.25	0.2652–3.1315 1.2300 ± 0.75	0.0632–0.9774 0.4465 ± 0.23
			0.5212 ± 0.60			
		BCF_W_	207.77–4399.61 1792.75 ± 1358.27	102.25–5303.62 1814.21 ± 1748.98	420.79–14,967.59 4391.97 ± 2962.07	13.84–2398.60 246.62 ± 424.87
			2061.39 ± 2383.09			
	B	P	16.89–120.04 49.67 ± 23.52	46.43–169.49 110.38 ± 34.88	27.22–134.79 79.20 ± 32.27	25.63–80.08 47.46 ± 15.14
			714.68 ± 37.63			
		BCF_B_	0.0175–2.22 0.5072 ± 0.55	0.0287–3.46 1.42 ± 1.16	0.3857–2.77 1.37 ± 0.71	0.1492–1.99 0.83 ± 0.47
			1.03 ± 0.86			
		BCF_W_	469.27–29,163.40 3626.89 ± 5641.92	2159.59–12,726.10 6461.84 ± 3094.85	1645.91–6989.95 3981.76 ± 1648.14	42.28–1513.40 375.09 ± 305.49
			3611.39 ± 3967.21			
	S	P	43.01–85.70 62.17 ± 14.23	39.01–102.68 66.82 ± 20.11	57.01–206.54 104.34 ± 44.68	10.00–70.27 34.06 ± 20.99
			66.85 ± 37.24			
		BCF_B_	0.6694–1.78 1.12 ± 0.36	0.4520–2.01 0.9852 ± 0.46	0.8359–3.26 1.79 ± 0.65	0.1318–1.05 0.4766 ± 0.30
			1.09 ± 0.66			
		BCF_W_	627.36–5909.00 2432.90 ± 1845.40	2933.10–70,158.80 10,306.37 ± 1815.65	3369.96–12,008.30 6014.73 ± 2400.19	21.00–630.48 215.14 ± 203.81
			4742.30 ± 9901.70			
Fe	NS	P	2.96–541.53 199.89 ± 204.39	65.01–616.59 208.14 ± 123.18	16.32–2946.99 1056.19 ± 850.94	46.44–4985.87 381.03 ± 889.57
			461.31 ± 718.53			
		BCF_B_	0.0001–0.1691 0.0168 ± 0.02	0.0031–0.0529 0.0152 ± 0.0125	0.0010–0.2993 0.0731 ± 0.0767	0.0030–0.2074 0.0217 ± 0.04
			0.0320 ± 0.05			
		BCF_W_	1.23–1694.00 370.14 ± 443.67	49.48–4101.20 524.91 ± 791.51	24.94–10,035.41 1754.27 ± 1980.97	29.51–2413.32 484.94 ± 529.89
			783.57 ± 1254.75			
	B	P	156.34–632.98 355.48 ± 112.80	198.01–1002.88 487.69 ± 207.04	72.08–5081.90 1658.29 ± 1405.45	87.11–695.86 331.00 ± 189.35
			708.12 ± 906.19			
		BCF_B_	0.0185–0.1030 0.0385 ± 0.02	0.0158–3.53 0.1568 ± 0.63	0.0073–0.5283 0.1763 ± 0.16	0.0049–2.99 0.9449 ± 1.02
			0.3291 ± 0.70			
		BCF_W_	162.09–2758.79 360.43 ± 326.26	227.70–3209.08 564.85 ± 627.11	157.37–4535.96 1813.98 ± 1480.39	32.82–3261.00 719.64 ± 792.59
			864.72 ± 1070.73			
	S	P	125.32–252.99 176.01 ± 39.04	256.00–1654.80 654.29 ± 409.43	393.01–6597.40 3742.4 ± 1860.3	108.81–1163.50 457.15 ± 345.08
			1257.50 ± 1739.10			
		BCF_B_	0.0088–0.0255 0.0137 ± 0.01	0.0131–6.92 1.44 ± 2.09	0.0338–0.4875 0.2921 ± 0.15	0.0067–1.51 0.4918 ± 0.55
			0.5611 ± 1.21			
		BCF_W_	153.41–609.57 364.94 ± 170.57	376.31–4001.80 1227.60 ± 987.62	1318.67–16,874.94 7230.87 ± 4190.34	99.17–2943.52 841.75 ± 832.42
			2416.30 ± 3554.50			
Mn	NS	P	95.63–637.51 245.13 ± 126.18	16.49–369.65 81.24 ± 69.17	31.43–883.94 148.99 ± 168.06	13.06–3955.12 270.44 ± 711.32
			186.45 ± 380.10			
		BCF_B_	0.5156–2.76 1.52 ± 0.66	0.0968–1.30 0.4800 ± 0.31	0.0901–4.96 1.03 ± 0.96	0.0681–14.00 1.38 ± 2.50
			1.08 ± 1.46			
		BCF_W_	177.98–20,701.61 2870.61 ± 4592.15	61.28–3252.45 608.91 ± 635.14	59.91–7864.23 998.85 ± 1398.14	90.51–21,392.88 1853.58 ± 3840.08
			1582.98 ± 3209.89			
	B	P	110.03–189.34 114.61 ± 20.37	128.09–196.99 166.63 ± 16.63	35.44–1731.87 339.43 ± 380.89	32.58–198.63 114.55 ± 51.16
			191.30 ± 211.55			
		BCF_B_	0.6932–7.32 2.84 ± 1.97	0.9175–4.30 2.26 ± 1.03	0.1126–20.84 2.82 ± 4.35	0.2788–35.58 11.25 ± 11.54
			4.79 ± 7.29			
		BCF_W_	389.10–1623.80 913.29 ± 352.49	233.95–1419.03 687.73 ± 342.06	43.64–6050.89 937.72 ± 1170.59	129.17–2254.06 867.17 ± 540.35
			851.48 ± 696.75			
	S	P	102.11–156.66 134.22 ± 18.73	132.66–175.41 156.74 ± 14.17	95.05–2155.80 505.61 ± 569.06	12.03–195.91 57.73 ± 60.57
			213.58 ± 334.35			
		BCF_B_	0.6215–1.28 0.9692 ± 0.23	0.6333–10.67 5.01 ± 4.09	0.5574–13.90 3.59 ± 3.74	0.1297–3.16 1.19 ± 0.84
			2.69 ± 3.28			
		BCF_W_	207.61–10,155.10 1426.48 ± 2369.06	514.55–2106.89 970.79 ± 437.83	361.19–7412.04 1958.03 ± 2107.42	22.93–3092.28 528.80 ± 847.89
			1221.03 ± 1738.64			

NS—Nysa Szalona River; B—Bystrzyca River; S—Strzegomka River; P—metal content in the plant; BCF_B_—metals bioaccumulation factor—bottom sediment; BCF_W_—metals bioaccumulation factor—water.

**Table 6:** Metal content (mg·kg^−1^) of reed canary grass in three main rivers over a four-year study cycle.

Metal	River		Index	2015	2016	2017	2018
				Min–Max $\bar{x}$ ± SD			
Cu	NS	1	P	6.52–123.58 65.04 ± 58.51	5.48–9.46 7.47 ± 1.99	2.33–8.88 5.52 ± 3.12	10.52–13.25 11.91 ± 1.29
				22.48 ± 38.32			
		2		4.55–296.64 150.56 ± 145.99	6.54–10.98 8.57 ± 2.03	6.42–14.99 10.58 ± 4.16	3.44–14.81 9.10 ± 5.56
				44.70 ± 95.28			
		1	BCF_B_	0.0300–2.56 1.29 ± 1.26	0.0218–0.1918 0.1066 ± 0.09	0.0596–0.2670 0.1606 ± 0.10	0.4695–0.5126 0.4927 ± 0.02
				0.5131 ± 0.79			
		2		0.0124–8.39 4.18 ± 4.17	0.0186–0.3014 0.1546 ± 0.14	0.4459–0.5360 0.4947 ± 0.04	0.1037–0.4351 0.2676 ± 0.16
				1.27 ± 2.68			
		1	BCF_W_	1423.63–38,603.06 18,138.83 ± 16,805.12	56.26–149.66 103.11 ± 46.04	664.66–765.89 721.41 ± 35.21	333.87–2045.72 1201.43 ± 818.19
				5041.19 ± 11,318.37			
		2		1263.28–57,011.29 28,629.02 ± 27,343.78	86.85–130.61 106.87 ± 19.74	1736.05–2918.31 2310.17 ± 544.12	54.04–458.50 237.39 ± 183.68
				7820.86 ± 18,223.44			
	B	1	P	7.41–10.42 8.91 ± 1.50	5.63–6.96 6.20 ± 0.58	5.10–8.91 6.94 ± 1.83	5.63–6.72 6.18 ± 0.54
				7.06 ± 1.67			
		2		12.44–12.53 12.48 ± 0.04	4.96–6.34 5.61 ± 0.65	13.25–19.93 16.39 ± 3.07	4.12–6.26 5.19 ± 1.07
				9.92 ± 5.01			
		1	BCF_B_	0.0415–0.7355 0.3808 ± 0.34	0.0268–0.4555 0.2383 ± 0.21	0.2915–1.54 0.9089 ± 0.62	0.5078–0.9361 0.7226 ± 0.21
				0.5627 ± 0.47			
		2		0.0264–2.77 1.36 ± 1.34	0.0107–1.14 0.5640 ± 0.55	0.3253–0.5954 0.4560 ± 0.13	0.0982–0.4772 0.2840 ± 0.18
				0.6665 ± 0.84			
		1	BCF_W_	4165.00–7412.30 5706.17 ± 1500.28	1025.45–4024.50 2490.01 ± 1446.69	1457.49–1579.14 1524.99 ± 42.73	63.21–72.62 68.86 ± 3.60
				2447.51 ± 2317.07			
		2		2304.33–2722.46 2516.33 ± 170.86	919.94–1441.41 1168.39 ± 233.11	1949.13–4634.65 3239.59 ± 1252.31	46.78–100.48 71.98 ± 24.41
				1749.07 ± 1379.54			
	S	1	P	10.20–12.86 11.47 ± 1.04	8.10–12.69 10.52 ± 2.11	14.41–22.68 18.44 ± 3.84	3.02–5.91 4.39 ± 1.34
				11.21 ± 5.51			
		2		13.01–13.99 13.52 ± 0.39	11.33–29.89 20.50 ± 9.10	9.34–15.55 12.45 ± 3.04	3.85–7.88 5.89 ± 1.97
				13.09 ± 7.12			
		1	BCF_B_	0.6326–0.9718 0.7956 ± 0.15	0.6109–0.8718 0.7527 ± 0.12	1.09–1.30 1.19 ± 0.09	0.2861–0.4684 0.3718 ± 0.08
				0.7775 ± 0.31			
		2		1.62–1.99 1.81 ± 0.15	1.41–4.25 2.82 ± 1.39	1.16–2.24 1.70 ± 0.53	0.2281–1.11 0.6708 ± 0.44
				1.75 ± 1.09			
		1	BCF_W_	3830.41–5589.87 4741.21 ± 769.29	3842.81–4519.43 4210.69 ± 239.62	6469.00–9448.13 7729.80 ± 1223.75	30.47–98.15 62.89 ± 32.16
				4186.15 ± 2829.10			
		2		1717.95–2151.91 1938.34 ± 167.33	1934.78–4395.09 3126.59 ± 1179.91	1355.33–3022.59 2182.29 ± 798.55	37.19–157.37 97.34 ± 59.45
				1836.14 ± 1311.52			
Cd	NS	1	P	0.5964–5.07 2.83 ± 2.23	0.5632–0.6898 0.6264 ± 0.06	0.8212–0.8997 0.8602 ± 0.04	0.0984–1.02 0.9991 ± 0.01
				1.33 ± 1.42			
		2		1.21–8.24 4.72 ± 3.51	0.6345–1.37 0.9548 ± 0.32	1.00–1.14 1.08 ± 0.06	1.02–1.13 1.09 ± 0.04
				1.96 ± 2.38			
		1	BCF_B_	0.2263–5.10 2.66 ± 2.43	0.2719–0.5904 0.4299 ± 0.1573	0.4336–0.6485 0.5343 ± 0.1085	0.8891–0.9650 0.9284 ± 0.03
				1.1379 ± 1.51			
		2		0.2503–8.74 4.49 ± 4.24	0.2466–0.9958 0.6224 ± 0.37	0.8036–0.8387 0.8197 ± 0.01	1.06–1.13 1.10 ± 0.02
				1.7559 ± 2.65			
		1	BCF_W_	993.02–2993.50 1662.95 ± 742.16	469.33–1371.00 853.40 ± 375.53	1026.50–8997.00 5021.48 ± 3942.86	32.19–1447.43 679.65 ± 650.08
				2054.37 ± 2690.25			
		2		4048.33–7487.55 5560.33 ± 1521.37	334.16–2462.20 1300.12 ± 966.96	1418.13–5010.00 2720.15 ± 1345.65	25.71–27.56 26.66 ± 0.63
				2401.82 ± 2344.83			
	B	1	P	0.7603–0.9544 0.8567 ± 0.09	0.7850–1.13 0.9481 ± 0.16	1.47–2.09 1.77 ± 0.27	0.1001–2.34 1.22 ± 0.12
				1.19 ± 0.68			
		2		1.00–1.12 1.06 ± 0.06	1.01–1.02 1.01 ± 0.01	1.19–1.50 1.35 ± 0.15	0.0355–0.7485 0.3916 ± 0.36
				0.9511 ± 0.40			
		1	BCF_B_	0.3617–0.5671 0.4635 ± 0.1015	0.3077–0.7835 0.5471 ± 0.24	1.4940–3.24 2.34 ± 0.82	0.1657–2.20 1.18 ± 1.01
				1.13 ± 1.00			
		2		0.2575–4.09 2.14 ± 1.88	0.2454–4.01 2.10 ± 1.86	0.6409–0.6796 0.6595 ± 0.02	0.0208–0.6454 0.3318 ± 0.31
				1.3088 ± 1.56			
		1	BCF_W_	1267.33–4772.00 2571.62 ± 1256.67	1308.33–2202.00 1745.96 ± 375.38	2323.33–15,433.00 8760.72 ± 6199.75	167.00–11,684.50 4636.95 ± 4633.66
				4428.81 ± 4771.88			
		2		717.36–1874.17 1260.47 ± 532.16	3350.67–5061.00 3933.42 ± 789.62	1495.38–15,007.00 7051.15 ± 5968.66	71.40–1497.00 703.13 ± 630.84
				3237.04 ± 3946.18			
	S	1	P	0.8623–0.9654 0.9136 ± 0.05	0.7521–0.8564 0.8043 ± 0.05	0.7521–1.71 1.2430 ± 0.46	0.2543–0.7455 0.4994 ± 0.24
				0.8611 ± 0.37			
		2		0.9613–1.07 1.01 ± 0.04	0.9641–0.9976 0.9807 ± 0.02	0.9945–1.23 1.11 ± 0.11	0.4105–0.9645 0.6885 ± 0.27
				0.9501 ± 0.22			
		1	BCF_B_	0.9978–1.30 1.150.15	0.8568–1.19 1.03 ± 0.17	0.7617–2.70 1.74 ± 0.95	0.2403–0.7725 0.5054 ± 0.26
				1.10 ± 0.67			
		2		1.39–1.71 1.56 ± 0.15	1.62–2.2085 1.91 ± 0.28	1.67–2.66 2.16 ± 0.48	0.6064–1.02 0.8074 ± 0.19
				1.61 ± 0.59			
		1	BCF_W_	1070.44–8644.00 4896.66 ± 3736.62	1423.50–7532.00 3899.49 ± 2688.94	1504.20–17,076.00 9378.89 ± 7688.31	5.36–21.79 13.36 ± 7.97
				4547.10 ± 5584.48			
		2		534.06–1481.29 989.06 ± 446.36	828.58–1935.40 1285.91 ± 445.10	720.18–1657.50 1125.24 ± 385.21	11.22–21.39 16.37 ± 4.27
				854.15 ± 617.60			
Ni	NS	1	P	0.6325–6.46 3.55 ± 2.91	5.00–5.57 5.28 ± 0.28	3.42–7.09 5.24 ± 1.81	5.33–8.99 7.20 ± 1.54
				5.32 ± 2.29			
		2		0.9621–14.58 7.75 ± 6.78	6.11–6.56 6.44 ± 0.16	4.76–9.99 7.30 ± 2.49	5.41–9.37 7.35 ± 1.89
				7.21 ± 3.76			
		1	BCF_B_	0.0090–0.0787 0.0437 ± 0.04	0.0087–0.0784 0.0430 ± 0.04	0.0662–0.1688 0.1165 ± 0.05	0.1558–0.2274 0.1935 ± 0.03
				0.0993 ± 0.07			
		2		0.0287–0.0399 0.0343 ± 0.01	0.0134–0.2001 0.1050 ± 0.09	0.1604–0.1841 0.1726 ± 0.01	0.1178–0.2225 0.1684 ± 0.05
				0.1201. ± 0.08			
		1	BCF_W_	3162.50–6458.70 5067.65 ± 1387.99	48.15–106.64 76.56 ± 28.20	822.19–1531.15 1180.13 ± 343.52	76.76–943.87 494.24 ± 417.01
				1704.64 ± 2116.60			
		2		291.55–10,378.29 5224.55 ± 4924.02	44.48–105.97 76.07 ± 29.51	933.06–1637.31 1272.91 ± 324.99	38.69–75.25 57.33 ± 16.78
				1657.72 ± 3251.38			
	B	1	P	2.10–4.04 3.07 ± 0.96	5.41–6.99 6.20 ± 0.78	6.12–13.44 9.74 ± 3.59	0.6914–3.53 2.11 ± 1.42
				5.28 ± 3.61			
		2		6.58–7.13 6.86 ± 0.27	4.68–8.89 6.65 ± 1.94	9.54–13.98 11.72 ± 2.17	0.1625–4.3487 2.2557 ± 2.09
				6.87 ± 3.80			
		1	BCF_B_	0.0061–0.2014 0.1021 ± 0.09	0.0205–0.2848 0.1513 ± 0.13	0.2701–1.0298 0.6475 ± 0.37	0.0464–0.1759 0.1109 ± 0.06
				0.2529 ± 0.31			
		2		0.0168–1.1192 0.5593 ± 0.54	0.0213–0.6688 0.3288 ± 0.31	0.2472–0.3278 0.2874 ± 0.04	0.0041–0.1882 0.0954 ± 0.09
				0.3177 ± 0.36			
		1	BCF_W_	2102.00–4037.00 2896.54 ± 815.11	3327.47–4513.17 3887.85 ± 524.19	1285.54–2986.44 2106.94 ± 816.65	6.64–32.51 19.56 ± 12.85
				2227.72 ± 1557.25			
		2		5480.38–6587.00 5744.16 ± 411.29	2930.44–3867.17 3401.78 ± 397.87	1644.91–3233.07 2418.98 ± 763.69	1.55–42.14 21.49 ± 19.91
				2896.60 ± 2107.68			
	S	1	P	4.42–5.53 4.90 ± 0.48	2.95–5.65 4.30 ± 1.34	8.66–31.67 20.06 ± 11.26	2.88–3.12 3.02 ± 0.09
				8.07 ± 8.97			
		2		5.45–6.01 5.84 ± 0.23	3.41–6.45 4.87 ± 1.44	9.12–9.56 9.29 ± 0.17	3.41–6.91 4.94 ± 1.54
				6.24 ± 2.09			
		1	BCF_B_	0.2073–0.2713 0.2356 ± 0.03	0.1305–0.2871 0.2073 ± 0.08	0.3874–1.57 0.9724 ± 0.58	0.1356–0.1427 0.1394 ± 0.01
				0.3886 ± 0.45			
		2		0.2774–0.3213 0.2935 ± 0.01	0.1645–0.3067 0.2331 ± 0.07	0.4367–0.5207 0.4762 ± 0.04	0.1749–0.3515 0.2511 ± 0.08
				0.3135 ± 0.11			
		1	BCF_W_	53.09–1086.19 564.33 ± 508.33	1063.91–1185.28 1123.15 ± 48.90	65.54–7256.98 3592.30 ± 3526.38	21.57–24.86 22.97 ± 1.28
				1325.68 ± 2244.52			
		2		43.35–1034.88 534.41 ± 488.99	972.19–1272.81 1111.70 ± 120.20	70.67–1731.72 882.16 ± 811.32	30.44–51.48 39.07 ± 8.69
				641.84 ± 625.55			
Pb	NS	1	P	29.55–32.53 31.05 ± 1.44	14.34–16.55 15.49 ± 1.05	8.87–12.32 10.58 ± 1.70	15.44–43.53 29.51 ± 13.78
				21.66 ± 11.25			
		2		45.05–46.64 45.74 ± 0.59	16.00–18.89 17.44 ± 1.30	13.12–25.96 19.37 ± 6.17	16.53–45.94 31.06 ± 14.30
				28.40 ± 13.73			
		1	BCF_B_	0.2361–1.01 0.6189 ± 0.38	0.1101–0.5334 0.3208 ± 0.21	0.2089–0.2209 0.2153 ± 0.01	0.3999–1.53 0.9617 ± 0.55
				0.5292 ± 0.46			
		2		0.1000–0.5801 0.3382 ± 0.24	0.0348–0.2533 0.1429 ± 0.11	0.3089–0.9094 0.5982 ± 0.29	0.4078–0.9346 0.6655 ± 0.25
				0.4362 ± 0.31			
		1	BCF_W_	596.99–29,659.80 14,658.69 ± 14,091.71	5699.86–5975.92 5812.52 ± 100.15	6826.54–10,267.67 8594.17 ± 1426.70	149.41–8807.83 4222.68 ± 4079.91
				8322.12 ± 8375.52			
		2		16,087.57–41,962.45 28,033.18 ± 11,745.09	4572.71–5554.24 5031.24 ± 388.23	6561.70–23,602.73 14,704.25 ± 7914.10	46.31–175.22 109.59 ± 62.52
				11,969.57 ± 12,796.99			
	B	1	P	3.52–3.87 3.69 ± 0.17	21.09–24.96 22.88 ± 1.55	19.07–21.99 20.49 ± 1.39	5.63–9.29 7.45 ± 1.82
				13.63 ± 8.33			
		2		4.52–8.53 6.52 ± 1.20	32.57–42.85 37.59 ± 4.85	14.00–23.89 18.95 ± 4.66	2.0020–9.2873 5.64 ± 3.64
				17.18 ± 13.50			
		1	BCF_B_	0.0013–0.0754 0.0382 ± 0.04	0.0174–0.4970 0.2516 ± 0.23	0.1918–0.4418 0.3158 ± 0.12	0.0821–0.1233 0.1017 ± 0.02
				0.1768 ± 0.17			
		2		0.0038–0.1155 0.0590 ± 0.06	0.0190–0.8283 0.4163 ± 0.40	0.0763–0.0861 0.0815 ± 0.01	0.0102–0.1388 0.0744 ± 0.06
				0.1578 ± 0.25			
		1	BCF_W_	3867.10–38,613.00 22,042.68 ± 11,725.19	7812.33–24,963.20 15,522.88 ± 7471.81	10,594.56–219,934.00 96,694.78 ± 93,143.62	2451.52–3869.75 3146.22 ± 604.92
				34,351.64 ± 59,657.12			
		2		471.17–8528.70 4498.75 ± 4025.72	20,356.13–23,385.17 22,059.91 ± 913.96	7001.25–23,894.30 14,793.09 ± 7440.88	770.04–4021.09 2349.69 ± 1588.02
				10,925.36 ± 9064.33			
	S	1	P	6.45–9.08 7.78 ± 1.27	7.52–8.91 8.15 ± 0.52	7.16–15.98 11.58 ± 4.19	3.91–7.52 5.70 ± 1.76
				8.30 ± 3.18			
		2		9.08–12.05 10.72 ± 1.34	8.09–14.65 11.39 ± 3.07	5.63–11.49 8.55 ± 2.89	8.13–10.94 9.44 ± 1.25
				10.02 ± 2.55			
		1	BCF_B_	0.1040–0.1436 0.1238 ± 0.02	0.1187–0.1339 0.1256 ± 0.01	0.1099–0.2563 0.1834 ± 0.07	0.0619–0.0997 0.0806 ± 0.02
				0.1283 ± 0.05			
		2		0.1859–0.3607 0.2758 ± 0.08	0.1587–0.2591 0.2089 ± 0.05	0.1158–0.3516 0.2332 ± 0.12	0.0949–0.2194 0.1540 ± 0.07
				0.2180 ± 0.10			
		1	BCF_W_	1864.03–11,348.50 6214.70 ± 4282.28	3008.56–12,733.14 7171.37 ± 4199.63	2752.65–15,645.00 8646.56 ± 5814.11	11.34–37.14 24.12 ± 12.71
				5514.19 ± 5314.33			
		2		1892.38–4818.08 3311.41 ± 1383.98	3842.36–6974.05 5339.08 ± 1442.70	2157.64–2571.68 2335.32 ± 165.63	32.92–37.79 36.19 ± 1.57
				2755.50 ± 2155.19			
Zn	NS	1	P	5.96–45.49 25.66 ± 19.69	46.00–52.65 49.49 ± 3.01	55.09–64.86 59.89 ± 4.67	36.09–54.52 45.35 ± 8.87
				45.09 ± 16.68			
		2		22.42–66.87 44.43 ± 21.95	48.12–66.91 57.56 ± 9.12	74.02–74.99 74.56 ± 0.41	40.41–56.65 48.52 ± 7.87
				56.27 ± 17.06			
		1	BCF_B_	0.0505–0.0902 0.0705 ± 0.0197	0.0523–0.7169 0.3821 ± 0.33	0.2652–0.3642 0.3147 ± 0.05	0.3723–0.5444 0.4591 ± 0.08
				0.3066 ± 0.23			
		2		0.0530–0.1812 0.1168 ± 0.06	0.0307–0.3918 0.2088 ± 0.18	0.8126–1.51 1.16 ± 0.34	0.5900–0.6212 0.6058 ± 0.01
				0.5223 ± 0.46			
		1	BCF_W_	207.77–2392.62 1281.57 ± 1053.38	214.77–3631.36 1898.25 ± 1681.41	420.79–4205.34 3095.83 ± 1270.18	230.55–2398.60 1300.29 ± 1068.89
				1893.99 ± 1488.68			
		2		911.28–4399.61 2647.15 ± 1729.75	291.36–4344.51 2307.41 ± 2012.97	3614.31–4933.39 4282.17 ± 629.29	161.94–248.12 204.94 ± 42.02
				2360.42 ± 1991.61			
	B	1	P	49.23–50.42 49.89 ± 0.53	70.00–125.94 97.77 ± 27.62	119.54–132.93 126.00 ± 6.39	26.14–70.24 48.34 ± 21.86
				80.50 ± 37.49			
		2		55.53–62.44 58.91 ± 3.38	98.11–136.50 117.43 ± 19.03	81.15–107.62 94.34 ± 12.89	29.31–51.77 40.49 ± 11.07
				77.79 ± 32.62			
		1	BCF_B_	0.0181–0.9612 0.4886 ± 0.47	0.0578–2.51 1.28 ± 1.22	1.1624–2.77 1.96 ± 0.79	0.2324–1.52 0.8727 ± 0.64
				1.15 ± 0.99			
		2		0.0274–1.42 0.7147 ± 0.69	0.0443–3.41 1.72 ± 1.67	0.3857–0.4457 0.4152 ± 0.03	0.1492–0.7739 0.4602 ± 0.31
				0.8268 ± 1.06			
		1	BCF_W_	1946.53–4337.12 3123.72 ± 1169.94	2602.36–10,075.50 6307.95 ± 3696.84	5559.95–6989.95 6270.12 ± 697.66	153.49–1513.40 422.49 ± 489.29
				4031.07 ± 3154.53			
		2		1559.79–3406.24 2481.04 ± 916.86	4694.05–6964.15 5833.19 ± 1117.42	4576.53–4775.70 4678.18 ± 78.88	42.28–335.91 188.34 ± 145.45
				3295.19 ± 2279.59			
	S	1	P	45.65–56.86 51.23 ± 5.46	70.01–96.87 83.28 ± 13.22	88.27–108.90 98.59 ± 10.00	10.00–38.36 24.18 ± 14.00
				64.32 ± 30.89			
		2		59.09–60.44 59.86 ± 0.59	66.02–102.68 84.38 ± 17.87	70.43–112.54 91.44 ± 20.89	17.03–52.89 35.11 ± 17.68
				67.70 ± 27.54			
		1	BCF_B_	0.7320–0.9009 0.8156 ± 0.08	1.05–1.54 1.29 ± 0.24	1.42–1.67 1.55 ± 0.12	0.1318–0.6073 0.3696 ± 0.24
				1.01 ± 0.49			
		2		1.249–1.78 1.50 ± 0.27	1.17–2.01 1.59 ± 0.41	2.14–2.34 2.24 ± 0.07	0.1988–1.05 0.6265 ± 0.42
				1.49 ± 0.66			
		1	BCF_W_	656.87–4704.34 2624.88 ± 1947.41	6108.96–70,157.80 5083.80 ± 29,164.60	4927.71–8198.44 6626.96 ± 1552.14	21.00–55.57 38.18 ± 16.81
				11,093.50 ± 20,286.70			
		2		627.36–3167.62 1891.42 ± 1245.68	3493.29–6075.48 4799.78 ± 1262.19	3369.96–5895.50 4631.17 ± 1250.16	45.62–290.42 166.61 ± 120.41
				2872.24 ± 2225.64			
Fe	NS	1	P	5.63–256.55 131.04 ± 125.40	120.00–263.56 191.98 ± 71.53	101.60–15,003.89 802.59 ± 700.83	326.74–350.99 338.70 ± 11.91
				366.08 ± 444.13			
		2		6.33–469.53 237.83 ± 231.49	113.53–270.45 192.01 ± 78.31	16.26–256.57 136.32 ± 120.04	86.09–369.68 227.86 ± 141.58
				332.71 ± 417.51			
		1	BCF_B_	0.0002–0.0177 0.0089 ± 0.0087	0.0072–0.0094 0.0083 ± 0.0011	0.0026–0.0994 0.0510 ± 0.05	0.0209–0.0239 0.0224 ± 0.01
				0.0227 ± 0.03			
		2		0.0003–0.0266 0.0134 ± 0.01	0.0065–0.1510 0.0108 ± 0.0043	0.0010–0.0989 0.0401 ± 0.04	0.0047–0.0212 0.0129 ± 0.01
				0.0193 ± 0.03			
		1	BCF_W_	21.11–251.25 136.11 ± 114.86	119.70–1048.74 576.86 ± 456.69	126.99–1501.24 813.36 ± 686.22	207.91–517.49 361.53 ± 153.39
				471.96 ± 492.12			
		2		6.57–958.03 480.73 ± 474.15	200.90–280.79 240.71 ± 39.33	24.94–2486.50 973.17 ± 1068.21	165.91–758.57 459.77 ± 292.11
				538.59 ± 659.53			
	B	1	P	365.41–452.99 408.99 ± 43.35	521.01–692.91 606.71 ± 85.63	686.25–5081.90 2883.98 ± 2197.45	87.11–652.64 369.85 ± 282.41
				1067.38 ± 1528.89			
		2		406.09–632.98 519.42 ± 113.10	201.34–769.99 485.52 ± 284.11	3251.47–4206.89 3729.08 ± 477.51	413.15–452.19 432.74 ± 19.41
				1291.69 ± 1435.87			
		1	BCF_B_	0.0332–0.0461 0.0397 ± 0.01	0.0473–0.0474 0.0474 ± 0.01	0.0427–0.4425 0.2426 ± 0.20	0.0048–1.25 0.6257 ± 0.62
				0.2388 ± 0.40			
		2		0.0357–0.1030 0.0693 ± 0.03	0.0253–0.0526 0.0389 ± 0.01	0.1782–0.5283 0.3532 ± 0.18	0.0378–1.70 0.8657 ± 0.8279
				0.3318 ± 0.54			
		1	BCF_W_	226.50–2758.79 671.64 ± 933.91	348.73–517.88 433.67 ± 84.26	472.34–4535.96 2447.62 ± 1975.95	32.82–322.23 177.47 ± 144.37
				932.57 ± 1413.08			
		2		411.40–511.87 462.39 ± 49.30	233.25–573.68 402.98 ± 169.53	3607.13–4288.67 3941.56 ± 334.12	437.79–1496.85 964.68 ± 526.57
				1442.56 ± 1494.56			
	S	1	P	125.32–222.88 174.04 ± 48.46	496.05–613.34 554.80 ± 58.43	4907.10–4963.90 4935.40 ± 27.99	144.23–1163.50 653.95 ± 509.40
				1579.50 ± 1962.80			
		2		221.03–252.99 237.13 ± 15.66	512.77–1654.80 1083.60 ± 570.77	2031.20–5006.40 3518.80 ± 1487.40	222.15–854.89 538.50 ± 316.02
				1344.50 ± 1525.60			
		1	BCF_B_	0.0088–0.0165 0.0126 ± 0.01	0.0338–2.02 1.03 ± 0.9934	0.3392–0.3969 0.3680 ± 0.03	0.0869–0.4774 0.2815 ± 0.1946
				0.4223 ± 0.63			
		2		0.0176–0.0255 0.0215 ± 0.01	0.0409–6.92 3.48 ± 3.44	0.2063–0.4613 0.3338 ± 0.13	0.0727–0.9682 0.5196 ± 0.45
				1.09 ± 2.22			
		1	BCF_W_	490.32–609.57 549.77 ± 58.65	1097.77–1677.41 1377.47 ± 278.28	11,633.22–16,874.94 14,123.69 ± 2481.96	404.46–2943.52 1669.22 ± 1262.44
				4430.04 ± 5783.56			
		2		390.58–557.27 473.06 ± 81.18	780.10–4001.80 2387.49 ± 1605.16	4726.72–9561.52 7069.07 ± 2319.89	601.37–1884.72 1246.37 ± 633.58
				2793.99 ± 2940.65			
Mn	NS	1	P	95.63–365.96 230.63 ± 134.88	40.05–153.63 97.03 ± 56.54	79.04–254.13 166.72 ± 87.37	156.43–290.79 223.47 ± 66.84
				179.47 ± 106.07			
		2		252.01–637.51 444.81 ± 192.49	36.45–155.91 96.10 ± 59.59	45.67–883.94 464.63 ± 418.87	123.52–296.45 210.00 ± 86.31
				303.89 ± 283.67			
		1	BCF_B_	0.5156–1.83 1.17 ± 0.66	0.2315–0.5782 0.4056 ± 0.1722	0.0901–1.35 0.7190 ± 0.63	1.01–1.63 1.32 ± 0.31
				0.9041 ± 0.61			
		2		1.40–1.96 1.68 ± 0.28	0.1922–0.5364 0.3643 ± 0.17	0.1423–4.96 2.55 ± 2.41	0.4939–1.64 1.07 ± 0.57
				1.42 ± 1.48			
		1	BCF_W_	567.56–2966.96 1741.59 ± 1164.02	244.83–1058.07 645.09 ± 398.86	633.85–1587.54 1094.89 ± 459.81	1354.12–1890.82 1611.82 ± 243.05
				1273.35 ± 797.64			
		2		2184.50–4381.52 3127.81 ± 949.03	233.86–1241.07 730.59 ± 494.20	269.77–7864.23 3935.16 ± 3660.68	831.26–2643.55 1739.42 ± 893.07
				2383.24 ± 2315.79			
	B	1	P	125.33–165.79 145.54 ± 20.17	165.44–193.95 179.58 ± 13.96	306.99–1731.87 1019.63 ± 712.04	64.00–193.95 128.92 ± 64.59
				368.42 ± 519.26			
		2		163.09–189.34 176.22 ± 12.93	163.00–166.94 154.12 ± 1.76	239.15–955.98 597.57 ± 358.08	32.57–148.96 90.58 ± 59.93
				257.37 ± 269.44			
		1	BCF_B_	0.9227–4.57 2.74 ± 1.81	1.22–4.30 2.75 ± 1.52	1.53–20.84 11.15 ± 9.62	0.29–21.47 10.88 ± 10.58
				6.87 ± 8.34			
		2		1.33–3.37 2.34 ± 1.01	1.29–2.6377 1.96 ± 0.67	1.92–2.1983 2.05 ± 0.14	0.31–29.79 13.65 ± 13.42
				5.00 ± 8.39			
		1	BCF_W_	389.10–1090.69 735.73 ± 345.71	237.03–531.98 453.23 ± 103.12	540.32–4111.76 2310.95 ± 1766.90	129.17–1310.00 610.18 ± 403.06
				1027.52 ± 1188.58			
		2		860.29–1307.91 1081.87 ± 220.69	238.71–875.25 554.02 ± 314.64	424.63–3706.82 1699.80 ± 1325.57	194.73–1613.91 873.63 ± 677.29
				1052.33 ± 875.20			
	S	1	P	1125.52–145.99 129.16 ± 16.49	152.56–156.96 154.64 ± 2.03	212.45–2155.80 1184.00 ± 971.47	12.03–195.91 103.98 ± 91.67
				392.95 ± 668.61			
		2		121.06–155.90 138.52 ± 17.14	165.06–169.88 167.62 ± 2.11	310.05–543.88 427.06 ± 116.65	25.33–153.57 89.49 ± 63.85
				205.67 ± 147.01			
		1	BCF_B_	0.7196–1.13 0.92 ± 0.20	1.12–8.60 4.86 ± 3.73	1.65–13.91 7.77 ± 6.12	7.75–130.36 68.98 ± 61.22
				3.77 ± 4.52			
		2		1.12–1.28 1.20 ± 0.08	1.22–9.39 5.30 ± 4.08	2.34–5.20 3.77 ± 1.42	1.16–3.16 2.12 ± 0.95
				3.10 ± 2.71			
		1	BCF_W_	420.79–1592.16 977.06 ± 553.53	514.55–2106.88 1279.85 ± 764.66	462.29–7412.04 3882.99 ± 3417.81	38.54–3092.28 1495.17 ± 1457.92
				1908.77 ± 2237.76			
		2		515.33–1177.46 846.75 ± 327.44	833.96–866.77 852.94 ± 12.24	1666.21–4476.28 3032.40 ± 1352.35	167.87–883.92 518.86 ± 349.48
				1312.74 ± 1232.65			

NS—Nysa Szalona River; B—Bystrzyca River; S—Strzegomka River; 1—river below its source; 2—river mouth to the reservoir; P—metal content in the plant; BCF_B_—metals bioaccumulation factor—bottom sediment; BCF_W_—metals bioaccumulation factor—water.

**Table 7:** Metals (mg·kg^−1^) and metal bioaccumulation factors relative to water (BCF_W_) and bottom sediment (BCF_B_) in reed canary grass in successive tributaries of the Strzegomka River in spring and autumn (mean values).

Site	Season	Index	Cu	Cd	Ni	Pb	Zn	Fe	Mn
Strzegomka below springs	s	P	10.30	0.71	5.06	6.38	72.26	1687.20	177.63
		BCF_B_	0.7833	0.7232	0.2326	0.10	1.13	0.1172	1.35
		BCF_W_	4037.81	1094.08	328.29	1970.11	2983.41	4041.98	1747.57
	a	P	12.11	1.02	11.08	10.23	56.38	1471.90	608.27
		BCF_B_	0.7717	1.49	0.5447	0.1561	0.8763	0.7275	6.19
		BCF_W_	4334.50	8000.12	2323.08	9058.27	19,203.50	4818.09	2069.96
Polska Woda	s	P	10.82	0.5710	4.74	7.67	89.80	549.32	133.34
		BCF_B_	0.8274	0.7061	0.2492	0.0883	1.04	0.0339	0.9330
		BCF_W_	1711.67	821.66	374.40	2373.17	3530.49	1643.79	1003.96
	a	P	9.86	0.7800	5.11	9.39	56.49	246.34	107.69
		BCF_B_	0.9206	1.17	0.2329	0.1407	3433.07	0.2610	2.82
		BCF_W_	1464.08	1056.72	745.95	5222.26	68.52	678.77	424.56
Sikorka	s	P	11.29	0.9400	4.62	7.12	67.79	1867.00	136.65
		BCF_B_	1.27	1.20	0.2134	0.0738	0.7040	0.1225	0.6572
		BCF_W_	1372.16	1381.80	355.95	1694.61	2287.88	1871.09	2446.56
	a	P	14.66	1.08	9.45	9.58	79.00	1163.40	219.54
		BCF_B_	1.36	1.51	0.3947	0.1325	1.16	0.8479	3.26
		BCF_W_	1581.53	1211.68	1822.96	8294.48	4369.11	2042.19	375.59
Czyżynka	s	P	9.48	1.07	5.82	7.41	63.46	1104.60	138.55
		BCF_B_	0.9433	1.26	0.2859	0.1195	1.01	0.0707	0.8605
		BCF_W_	1776.63	1319.54	556.17	1905.26	2270.06	1168.16	899.52
	a	P	14.18	0.8900	7.67	9.54	47.89	1795.70	202.75
		BCF_B_	1.34	1.40	0.3397	0.2220	1.18	1.23	4.64
		BCF_W_	1819.77	1201.47	1436.06	5560.19	3600.86	2310.72	617.05
Strzegomka mouth to the reservoir	s	P	12.12	0.8611	6.26	8.50	81.95	1648.80	196.23
		BCF_B_	1.68	1.33	0.3279	0.1707	1.65	0.1482	1.50
		BCF_W_	1795.60	1165.92	347.92	2090.33	3218.96	3110.24	1141.93
	a	P	14.06	1.04	6.21	11.54	53.45	1040.30	215.11
		BCF_B_	1.82	1.88	0.2989	0.2652	1.33	2.03	4.69
		BCF_W_	1876.68	542.38	935.75	3420.68	2525.52	2477.76	1483.55

s—spring, a—autumn, P—metal content in the plant; BCF_B_—metals bioaccumulation factor—bottom sediment; BCF_W_—metals bioaccumulation factor—water

**Table 8:** Metals (mg·kg^−1^) and metal bioaccumulation factors relative to water (BCF_W_) and bottom sediment (BCF_B_) in reed canary grass in successive tributaries of the Bystrzyca River in spring and autumn (mean values).

Site	Season	Index	Cu	Cd	Ni	Pb	Zn	Fe	Mn
Bystrzyca below springs	s	P	6.22	1.34	4.79	14.85	83.41	415.17	165.77
		BCF_B_	0.4935	1.27	0.2322	0.2088	1.21	0.0321	0.9941
		BCF_W_	3174.44	6717.68	2352.17	57,406.38	5468.21	535.64	458.81
	a	P	7.89	1.05	5.77	12.41	77.60	1719.59	571.06
		BCF_B_	0.6319	0.9984	0.2737	0.1448	1.08	0.4456	12.76
		BCF_W_	1720.57	2139.94	2103.28	11,296.89	2593.93	1329.51	1596.22
Złoty Potok	s	P	8.27	1.02	4.85	16.39	94.12	545.72	169.20
		BCF_B_	0.6213	1.80	0.4570	0.1977	1.15	0.0476	1.14
		BCF_W_	2131.25	5943.15	2201.22	12,142.10	4140.68	518.16	503.09
	a	P	7.63	0.7564	3.30	12.18	70.20	687.68	268.28
		BCF_B_	0.5351	1.45	0.3210	0.1512	0.8652	0.4259	7.66
		BCF_W_	1576.38	1742.05	1364.94	7337.48	8874.15	533.96	992.46
Kłobia	s	P	8.46	0.9388	5.30	17.05	89.21	1083.46	150.64
		BCF_B_	0.3990	1.06	0.2667	0.2048	0.9493	0.1175	0.9691
		BCF_W_	3596.49	6225.83	2482.41	12,210.10	3805.27	951.33	768.89
	a	P	7.16	0.7282	3.72	10.24	67.12	653.10	170.42
		BCF_B_	0.7448	0.9555	0.1969	0.11	0.8091	0.7957	8.92
		BCF_W_	1603.51	1966.61	1899.68	6915.97	3220.96	594.54	549.43
Otłuczyna	s	P	8.45	0.8967	4.32	17.19	100.09	594.76	138.79
		BCF_B_	0.4574	1.63	0.3814	0.2923	1.68	0.0579	0.8988
		BCF_W_	4623.78	4974.69	2067.74	14,035.75	4705.91	525.13	799.02
	a	P	5.01	0.7996	4.04	14.61	50.04	596.33	159.39
		BCF_B_	0.2959	1.40	0.3957	0.2483	0.8194	0.3840	6.03
		BCF_W_	1013.38	2395.95	2220.69	8939.17	2274.85	882.01	521.66
Potok Marcowy Duży	s	P	6.22	0.6389	7.46	12.69	70.77	309.96	104.91
		BCF_B_	0.6158	0.9667	0.5000	0.2992	1.56	0.0328	0.8144
		BCF_W_	2487.10	2746.72	3399.60	9019.40	3425.44	368.81	839.35
	a	P	4.83	0.8333	4.96	8.98	43.05	412.27	149.73
		BCF_B_	0.2013	0.8122	0.1518	0.0984	0.6191	0.3439	6.67
		BCF_W_	1451.23	4345.64	2644.82	5215.77	2467.25	1475.80	1051.07
Złota Woda	s	P	4.70	0.5986	3.76	12.81	56.13	207.40	115.94
		BCF_B_	0.3904	1.45	0.2851	0.2718	1.17	0.0235	0.9695
		BCF_W_	1402.44	4050.17	1946.31	10,419.69	2280.19	352.68	1216.42
	a	P	10.40	0.7948	3.98	11.19	59.70	1106.74	220.01
		BCF_B_	0.5373	0.5637	0.0665	0.1115	0.9112	0.8570	7.63
		BCF_W_	1830.70	3773.95	1502.36	8345.79	2267.77	2060.09	1018.47
Rybna	s	P	8.12	0.7896	5.85	14.84	67.48	670.31	136.13
		BCF_B_	0.7871	1.76	0.5468	0.2702	1.25	0.0805	0.8271
		BCF_W_	1523.15	4205.99	3029.93	11,317.14	2792.60	1124.01	1197.50
	a	P	8.16	0.7172	2.89	10.09	55.41	365.65	137.14
		BCF_B_	1.09	0.6139	0.1091	0.1167	1.02	0.4371	8.65
		BCF_W_	1906.24	2753.07	1463.51	5727.57	2388.47	279.38	539.23
Jaworzynik	s	P	7.96	0.8315	5.92	16.61	70.24	270.17	148.86
		BCF_B_	0.6727	0.9337	0.3613	0.3307	1.35	0.6132	1.67
		BCF_W_	1760.75	3752.18	2880.87	13,096.07	3566.47	381.96	1102.57
	a	P	10.72	0.7510	4.91	11.41	73.74	840.96	239.53
		BCF_B_	0.6312	0.8346	0.1875	0.0756	0.8429	0.5640	11.69
		BCF_W_	2162.08	2489.62	2193.52	7099.72	3982.27	809.70	404.88
Walimianka	s	P	7.67	0.8641	6.97	13.46	75.83	284.44	98.98
		BCF_B_	0.3631	0.6287	0.2113	0.1269	0.7420	0.0285	1.49
		BCF_W_	1980.58	5970.66	3383.59	12,188.28	3891.11	423.20	742.38
	a	P	10.98	1.83	5.91	15.99	73.81	815.24	166.56
		BCF_B_	0.2889	1.99	0.2231	0.1940	0.9626	0.6319	6.10
		BCF_W_	1887.30	2857.64	3249.24	9677.82	3491.98	1262.78	623.38
Bystrzyca mouth to the reservoir	s	P	11.12	1.06	7.52	17.54	87.69	1306.91	328.75
		BCF_B_	1.22	2.32	0.5628	0.2878	1.49	0.1568	1.21
		BCF_W_	2167.43	4611.22	2968.02	11,986.16	3362.71	1340.07	1335.52
	a	P	8.71	0.8433	6.22	16.82	67.89	1276.47	185.99
		BCF_B_	0.1162	0.3019	0.0727	0.0278	0.1665	0.5068	8.79
		BCF_W_	1330.72	1862.86	2825.19	9864.57	3227.67	1545.73	769.14

s—spring, a—autumn, P—metal content in the plant; BCF_B_—metals bioaccumulation factor—bottom sediment; BCF_W_—metals bioaccumulation factor—water

**Table 9:** Metals and metal bioaccumulation factors relative to water (BCF_W_) and bottom sediment (BCF_B_) in reed canary grass in successive tributaries of the Nysa Szalona River in spring and autumn (mean values).

Site	Season	Index	Cu	Cd	Ni	Pb	Zn	Fe	Mn
Nysa Szalona below springs	s	P	36.51	1.89	3.82	19.26	38.37	174.43	188.77
		BCF_B_	0.8205	1.75	0.0801	0.5376	0.3614	0.0090	0.8776
		BCF_W_	9424.22	2944.20	1506.79	6159.87	1516.03	424.19	1488.53
	a	P	8.46	0.7734	6.81	24.06	51.82	557.73	170.16
		BCF_B_	0.2056	0.5259	0.1186	0.5208	0.2518	0.0363	0.9306
		BCF_W_	658.18	1164.54	1902.50	10,484.17	2271.94	519.73	1058.16
Kocik	s	P	48.63	2.95	5.66	34.43	35.16	105.77	131.99
		BCF_B_	1.50	2.98	0.1781	1.03	0.5112	0.0081	1.11
		BCF_W_	8740.48	3175.67	561.45	8032.97	900.15	287.88	1241.85
	a	P	7.00	0.7727	7.07	14.51	50.96	502.20	99.92
		BCF_B_	0.1691	0.4732	0.0901	0.1804	0.2915	0.0122	0.6130
		BCF_W_	892.75	1649.43	2728.70	8615.97	3713.70	495.16	604.44
Ochodnik	s	P	32.25	1.84	3.64	13.77	26.05	103.18	77.94
		BCF_B_	1.22	2.13	0.1099	0.7081	0.4611	0.0059	0.5096
		BCF_W_	10,944.88	1786.49	1445.85	4625.74	671.58	465.41	663.85
	a	P	8.37	0.8827	6.87	13.03	38.57	73.12	489.52
		BCF_B_	0.2151	0.4929	0.0668	0.2247	0.2314	0.0251	0.4797
		BCF_W_	808.44	1162.74	2012.21	6181.79	2112.21	468.08	135.31
Sadówka	s	P	31.44	1.66	5.28	26.95	26.86	153.60	91.67
		BCF_B_	2.11	5.30	0.2184	1.58	0.4366	0.0083	0.5537
		BCF_W_	4299.59	1624.59	1373.36	6138.93	464.15	627.35	918.26
	a	P	9.59	0.9681	7.35	13.21	41.98	957.77	139.19
		BCF_B_	0.3769	0.8065	0.1164	0.4682	0.4419	0.0547	0.8468
		BCF_W_	919.14	1768.17	2226.14	6942.56	2519.24	894.02	708.54
Czyściel	s	P	35.16	2.76	6.26	23.75	52.77	1727.19	1084.49
		BCF_B_	1.36	6.56	0.2214	1.38	0.7647	0.0703	3.99
		BCF_W_	6166.11	2966.49	911.38	6187.23	1241.38	872.83	5861.57
	a	P	11.02	0.99	8.06	13.80	61.31	1016.12	157.43
		BCF_B_	0.5367	0.8924	0.2279	0.3162	0.7770	1.28	0.1023
		BCF_W_	1366.99	1329.11	1580.58	6631.28	3562.12	1247.23	1275.31
Radynia	s	P	34.50	3.28	5.45	19.28	34.61	243.87	136.47
		BCF_B_	0.9045	5.63	0.2554	1.63	0.6668	0.0135	0.9791
		BCF_W_	7748.10	1875.02	494.26	6204.27	1108.77	173.24	329.66
	a	P	11.98	1.048	8.59	17.59	66.74	146.58	596.40
		BCF_B_	0.5168	0.9022	0.3013	0.6484	0.8925	0.0709	1.43
		BCF_W_	884.26	1759.90	1887.84	7592.92	3481.09	1145.74	6038.72
Nysa Mała	s	P	50.77	2.51	4.89	22.54	35.12	125.41	183.54
		BCF_B_	2.20	3.12	0.1716	1.30	0.6995	0.0205	1.32
		BCF_W_	13,980.43	3764.47	721.65	7320.76	872.36	589.14	2047.10
	a	P	6.68	1.08	8.29	14.28	52.31	376.44	143.59
		BCF_B_	0.2606	0.8715	0.1440	0.3690	0.3429	0.0379	1.32
		BCF_W_	966.50	3402.81	2314.30	6954.34	2696.28	1082.02	1066.88
Puszówka	s	P	59.47	3.09	5.24	24.67	37.66	260.57	162.69
		BCF_B_	1.64	3.05	0.1348	1.16	0.8649	0.0157	1.12
		BCF_W_	6938.02	3324.72	694.19	6799.53	927.84	216.59	939.19
	a	P	10.03	0.8572	7.84	15.65	54.17	627.12	107.78
		BCF_B_	0.4917	0.6968	0.1112	0.3349	0.3291	0.0557	0.9026
		BCF_W_	705.87	3181.92	2964.19	7768.64	2822.79	1820.98	1051.78
Jawornik	s	P	41.91	2.29	3.29	21.25	27.93	172.52	140.59
		BCF_B_	1.56	4.89	0.1133	0.4834	0.5384	0.0095	0.9221
		BCF_W_	3826.07	1605.25	346.08	5896.69	598.35	43.05	888.79
	a	P	9.11	0.8470	9.49	22.20	65.65	525.28	111.21
		BCF_B_	0.4925	0.5718	0.1328	0.4469	0.7497	0.0381	0.6697
		BCF_W_	763.88	3043.43	3648.54	9100.88	5875.10	1562.28	5067.93
Księginka	s	P	42.96	1.84	5.09	17.22	24.85	155.77	169.68
		BCF_B_	2.06	2.03	0.1733	0.5313	0.5833	0.0102	1.40
		BCF_W_	4630.89	1138.85	589.89	7565.16	471.94	182.38	1002.67
	a	P	10.19	0.8530	12.48	21.93	56.08	972.19	153.43
		BCF_B_	0.3443	0.4470	0.2115	0.5538	0.6312	0.0815	1.24
		BCF_W_	905.80	1368.26	3838.03	13,660.08	3746.98	4306.72	1407.27
Starucha	s	P	56.91	1.43	5.02	41.06	37.91	156.50	127.29
		BCF_B_	2.88	5.00	0.1689	0.6327	0.9066	0.0065	0.9767
		BCF_W_	16,282.90	1753.65	451.39	16,076.21	1212.12	78.55	577.34
	a	P	15.87	1.12	9.50	30.96	52.55	613.21	195.21
		BCF_B_	0.3554	0.7379	0.1420	0.5620	0.2910	0.0597	1.40
		BCF_W_	1374.50	2033.63	3201.54	13,961.11	3230.41	942.94	207.72
Rowiec	s	P	70.94	2.25	5.84	21.23	41.04	242.25	136.60
		BCF_B_	3.66	2.01	0.1996	0.7787	0.5844	0.0239	0.4243
		BCF_W_	20,728.49	2710.57	1690.98	11,299.22	931.04	185.35	1014.34
	a	P	12.09	1.02	9.62	28.89	24.41	668.89	102.55
		BCF_B_	0.2083	0.6039	0.1216	0.3819	0.1484	0.0419	0.6544
		BCF_W_	927.63	3409.07	2398.85	16,850.92	2001.01	868.68	1009.96
Męcinka	s	P	105.33	2.34	4.17	21.97	32.13	188.22	260.75
		BCF_B_	2.38	2.24	0.1056	0.4697	0.3827	0.0098	0.8907
		BCF_W_	19,305.28	3060.20	312.06	7877.71	1154.99	319.38	1963.53
	a	P	8.99	0.9406	9.28	28.89	57.52	539.16	120.20
		BCF_B_	0.1519	0.3449	0.0827	0.3536	0.3018	0.0212	0.6379
		BCF_W_	705.49	2702.23	2699.49	13,665.86	2894.48	1043.47	988.42
Nysa Szalona mouth to the reservoir	s	P	79.27	2.75	4.39	26.56	46.56	154.83	240.61
		BCF_B_	2.3199	2.9099	0.1319	0.5313	0.6728	0.0085	0.7831
		BCF_W_	14,478.94	2848.01	335.22	11,093.39	1278.15	185.05	1602.91
	a	P	10.14	1.17	10.03	30.24	65.98	510.58	367.16
		BCF_B_	0.2291	0.6019	0.1083	0.3412	0.3737	0.0301	2.0469
		BCF_W_	1162.78	1955.63	2980.21	12,845.75	3442.69	892.13	3163.58

s—spring, a—autumn, P—metal content in the plant; BCF_B_—metals bioaccumulation factor—bottom sediment; BCF_W_—metals bioaccumulation factor—water

**Table 10:** Spearman’s correlation of metal content in reed canary grass and water reaction, study site, season and year. Differences statistically significant at *p* ≤ 0.05 marked with red.

Ni	0.13										
Cd	0.34	0.22									
Pb	0.23	0.36	0.45								
Zn	0.05	0.30	0.03	0.04							
Mn	0.30	0.19	0.25	0.26	0.26						
Fe	−0.03	0.39	−0.06	−0.08	0.56	0.41					
pH	0.05	0.00	−0.27	−0.31	−0.10	−0.13	0.05				
site	0.03	0.19	0.18	0.29	−0.13	−0.01	−0.10	−0.07			
river	0.31	0.15	0.04	−0.03	−0.14	−0.10	−0.10	0.41	−0.13		
season	−0.06	0.24	−0.10	−0.09	0.10	0.08	0.33	0.25	0.00	0.00	
year	−0.14	0.01	−0.21	−0.19	−0.04	−0.19	0.14	0.37	0.00	0.00	0.00
	Cu	Ni	Cd	Pb	Zn	Mn	Fe	pH	site	river	season

**Table 11:** Metal pollution index (MPI) of aquatic plants, bottom sediments and water.

	Nysa Szalona			Bystrzyca			Strzegomka		
	Plant	Sediment	Water	Plant	Sediment	Water	Plant	Sediment	Water
	41.87	277.26	0.0903	31.81	189.41	0.0396	36.40	50.46	0.0826
pollution degree	high	highest	no effect	high	highest	no effect	high	high/very high	no effect

**Table 12:** Metal pollution index (MPI) of aquatic plants—mouth, sources in subsequent years.

	Nysa Szalona		Bystrzyca		Strzegomka	
	Source	Mouth	Source	Mouth	Source	Mouth
2015	16.71	28.90	15.70	22.54	17.09	20.72
pollution degree	moderate	high	moderate	high	moderate	high
2016	15.87	18.07	26.06	26.85	21.21	27.44
pollution degree	moderate		high			
2017	18.22	24.89	44.95	49.74	53.29	38.08
pollution degree	moderate	high			very high	high
2018	25.48	22.00	12.13	8.63	10.24	14.39
pollution degree	high		moderate			

**Table 13:** Enrichment factor (EF) in reed canary grass from the Bystrzyca River.

		Cu	Cd	Ni	Pb	Zn	Fe	Mn
Bystrzyca and tributaries	whole	12.86	7.03	4.33	76.91	14.41	7.32	49.51
	spring	208.43	115.48	79.68	1413.89	265.62	362.01	185.72
	autumn	6.79	3.66	1.99	35.44	6.62	8.17	16.73
tributaries	whole	11.46	6.11	3.75	67.91	12.74	5.59	37.47
	spring	330.61	129.76	82.69	793.15	286.31	868.54	184.04
	autumn	9.73	3.34	2.91	24.49	5.71	12.19	5.74
Bystrzyca below sources	whole	14.25	11.64	5.55	93.92	20.11	17.53	92.74
	spring	286.54	296.67	114.86	2335.42	475.51	450.83	328.71
	autumn	8.14	5.21	3.10	43.72	9.91	41.83	25.36
Bystrzyca outlet to the reservoir	whole	41.97	19.32	15.13	248.15	40.73	128.83	46.87
	spring	118.32	54.20	41.65	637.09	115.46	327.77	150.56
	autumn	22.99	10.70	8.55	151.62	22.18	79.45	21.14

**Table 14:** Enrichment factor (EF) in reed canary grass from the Nysa Szalona River.

		Cu	Cd	Ni	Pb	Zn	Fe	Mn
Nysa Szalona and tributaries	whole	33.42	8.54	3.79	81.88	5.94	4.74	21.39
	spring	1440.77	313.76	70.28	2261.97	122.05	185.42	267.61
	autumn	14.42	8.46	6.54	121.20	9.25	15.48	13.93
tributaries	whole	31.52	8.17	3.67	76.65	5.54	11.66	7.86
	spring	1462.44	324.74	74.68	2356.84	122.19	205.29	278.86
	autumn	5.42	2.39	2.37	35.07	3.42	8.13	2.92
Nysa Szalona below sources	whole	45.02	12.80	5.55	148.09	11.18	17.28	15.47
	spring	1519.64	378.06	82.76	2736.66	197.64	171.13	338.19
	autumn	8.68	3.81	3.64	84.28	6.58	13.49	7.52
Nysa Szalona outlet to the reservoir	whole	52.65	11.09	4.42	114.20	8.20	9.24	15.41
	spring	1268.06	211.41	36.55	1450.43	92.17	58.38	165.67
	autumn	6.20	3.44	3.19	63.13	4.99	7.36	9.66

**Table 15:** Enrichment factor (EF) in reed canary grass from the Strzegomka River.

		Cu	Cd	Ni	Pb	Zn	Fe	Mn
Strzegomka and tributaries	whole	14.68	5.29	4.24	36.87	10.22	6.22	66.88
	spring	280.40	103.53	71.49	657.66	241.13	174.87	839.28
	autumn	8.21	2.93	2.61	21.73	4.59	7.37	17.06
tributaries	whole	11.30	4.13	3.13	27.82	8.04	25.48	6.49
	spring	228.89	122.84	62.23	1148.85	216.59	230.89	458.38
	autumn	5.07	2.24	2.22	32.78	3.20	2.73	7.59
Strzegomka below sources	whole	23.37	8.42	8.75	59.09	16.59	35.26	77.62
	spring	17.71	120.88	93.30	771.58	316.79	270.86	1408.89
	autumn	364.89	5.26	6.19	37.51	7.49	37.26	28.12
Strzegomka outlet to the reservoir	whole	13.01	9.96	7.13	75.06	18.38	19.43	69.55
	spring	206.01	70.25	55.38	493.22	172.38	143.57	660.60
	autumn	16.49	5.86	3.79	46.21	7.76	28.76	10.86

## Supplementary Materials

The following supporting information can be downloaded at: https://www.mdpi.com/article/10.3390/ijerph19137779/s1, Table S1. Characteristics of the rivers under examination [13,14,15,19,20].
