# Supplementary material for: Possibility of Metal Accumulation in Reed Canary Grass (Phalaris arundinacea L.) in the Aquatic Environment of South-Western Polish Rivers
Source: Int J Environ Res Public Health. 2022 Jun 24;19(13):7779. doi: 10.3390/ijerph19137779 (PMC9266212; doi:10.3390/ijerph19137779)
Supplement: Supplementary file 1 [file ijerph-19-07779-s001.zip › ijerph-1740901-supplementary.pdf]

Table S1. Characteristics of the rivers under examination [13–15,19,20].

	Nysa Szalona	Strzegomka	Bystrzyca
Watershed	3rd order– a right-bank tributary of the Kaczawa river	2nd order – a left-bank tributary of the Bystrzyca	2nd order – a left-bank tributary of the Odra
Length (km)/ Catchment area (km^2)	51.00 / 443.10	74.70 / 555.00	95.20 / 1767.80
Springs, altitude m above sea level	Mount Pustelnik<br>628.00	Trójgarb<br>692.00	The Suche and Sowie Mountains<br>618.00
Estuary - dam reservoir/km of river/function/year of commissioning/reservoir location m.a.s.l./reservoir type/ catchment area above the dam reservoir (km^2)	Słup<br>8.20<br>Retention (flood wave reduction), water supply and municipal water supply for the region of Legnica<br>1984<br>165-257<br>Lowland<br>374.81	Dobromierz<br>62.00<br>Retention (flood wave reduction), water supply and municipal water supply for the region of Świebodzice<br>1988<br>300-423<br>lowland and upland<br>70.32	Lubachów<br>78.00<br>Retention (flood wave reduction), energy, water supply and municipal water supply for the region of Dzierżoniów<br>1918<br>400-500<br>Upland<br>130.69
Exploitation / pollution	Agricultural and forest areas, grasslands, sewage treatment plants (Wolbromek, Jawor), aggregate mine, highways, larger localities Bolków, Jawor	Agricultural areas, grasslands, loose rural buildings, sewage collection unorganized, larger locality Stare Bogaczowice	Agricultural and forest areas, grasslands, sewage treatment plant and landfill (Jugowice), larger cities Głuszyca, Jugowice, Zagórze Śląskie
Tributaries above the reservoir: left-bank	Męcinka, Rowiec, Starucha, Jawornik, Puszówka, Nysa Mała, Kamiennik	Sikorka	Otłuczyna,<br>Złota Woda,<br>Rybna
right-bank	Ochodnik, Sadówka, Czyściel, Parowa, Kocik	Polska Woda, Czyżynka	Złoty Potok, Kłobia, Potok Marcowy Duży, Jaworzynik, Walimianka
Soil in the area	Podzolic soils, brown soils, alluvial soils	Podzolic soils, leached and acid brown soils	Podzolic soils, leached brown soils, deluvial deposits
